# Distinct Traits of Structural and Regulatory Evolutional Conservation of Human Genes with Specific Focus on Major Cancer Molecular Pathways

**DOI:** 10.3390/cells12091299

**Published:** 2023-05-02

**Authors:** Galina Zakharova, Alexander Modestov, Polina Pugacheva, Rijalda Mekic, Ekaterina Savina, Anastasia Guryanova, Anastasia Rachkova, Semyon Yakushov, Andrei Alimov, Elizaveta Kulaeva, Elena Fedoseeva, Artem Kleyman, Kirill Vasin, Victor Tkachev, Andrew Garazha, Marina Sekacheva, Maria Suntsova, Maksim Sorokin, Anton Buzdin, Marianna A. Zolotovskaia

**Affiliations:** 1 World-Class Research Center “Digital Biodesign and Personalized Healthcare”, Sechenov First Moscow State Medical University, Moscow 119991, Russia; 2 Laboratory of Clinical and Genomic Bioinformatics, I.M. Sechenov First Moscow State Medical University, Moscow 119991, Russia; 3 Laboratory for Translational Genomic Bioinformatics, Moscow Institute of Physics and Technology, Dolgoprudny 141701, Russia; 4 Oncobox Ltd., Moscow 121205, Russia; 5 Omicsway Corp., Walnut, CA 91789, USA; 6 Laboratory of Systems Biology, Shemyakin-Ovchinnikov Institute of Bioorganic Chemistry, Moscow 117997, Russia; 7 PathoBiology Group, European Organization for Research and Treatment of Cancer (EORTC), 1200 Brussels, Belgium

**Keywords:** cancer molecular pathway, retroelements, RetroSpect, retrotransposons, epigenetics, gene expression regulation, evolution of protein-coding genes, human genome evolution, pathway analysis

## Abstract

The evolution of protein-coding genes has both structural and regulatory components. The first can be assessed by measuring the ratio of non-synonymous to synonymous nucleotide substitutions. The second component can be measured as the normalized proportion of transposable elements that are used as regulatory elements. For the first time, we characterized in parallel the regulatory and structural evolutionary profiles for 10,890 human genes and 2972 molecular pathways. We observed a ~0.1 correlation between the structural and regulatory metrics at the gene level, which appeared much higher (~0.4) at the pathway level. We deposited the data in the publicly available database RetroSpect. We also analyzed the evolutionary dynamics of six cancer pathways of two major axes: Notch/WNT/Hedgehog and AKT/mTOR/EGFR. The Hedgehog pathway had both components slower, whereas the Akt pathway had clearly accelerated structural evolution. In particular, the major hub nodes Akt and beta-catenin showed both components strongly decreased, whereas two major regulators of Akt TCL1 and CTMP had outstandingly high evolutionary rates. We also noticed structural conservation of serine/threonine kinases and the genes related to guanosine metabolism in cancer signaling: GPCRs, G proteins, and small regulatory GTPases (Src, Rac, Ras); however, this was compensated by the accelerated regulatory evolution.

## 1. Introduction

In the last decade, the concept of molecular evolution has been reshaped by contemporary analytic approaches curating big amounts of genetic and molecular data [1]. The assessment of gene expression variability is one of the most actively developing topics in this field [2], which helps to investigate the evolution of the whole gene network [3,4,5]. One of the major topics in the field is finding the hot spots in molecular evolution and an overall estimation of evolutionary rates for individual genes, molecular pathways, and whole genomes [6].

The evolutionary rates can be assessed directly for both structural and regulatory changes in the protein-coding genes. The structural component can be quantitated by measuring the ratio of non-synonymous to synonymous nucleotide substitutions in the protein-coding sequence, called the dN/dS approach [7]. This makes it possible to analyze the conservation of the functional gene sequences in a quantitative way at a high throughput and to identify genes with the most and the least quickly evolving protein structures.

In turn, the regulatory changes make another dimension of gene evolution. Even in closely related species with very similar genome sequences, gene expression patterns can be very different. This divergence in gene expression regulation can play an important role in speciation and is considered one of the main sources of phenotypic variability between species [8]. This component was problematic to measure until recently when we proposed the RetroSpect method of measuring the rate of gene regulatory evolution as the proportion of transposable elements which were domesticated by the host genome and used as the regulatory elements for a gene of interest [9].

Transposable elements are selfish genome sequences that are able to self-reproduce in the host genomes. They harbor a number of regulatory sequences that can affect the epigenetic and transcriptional control of a gene [10]. Retroelements (REs) are transposable elements that proliferate through the reverse transcription of their RNA products. REs are thought to be the only class of transposable elements that were transpositionally active in the human lineage after mammalian radiation [11]. Upon insertion into a new site of the genome, RE copy is normally epigenetically silenced because its potential transcriptional and regulatory activities can be deleterious for the regulatory profile of the neighboring genes [12]. However, this can change over time when the removal of epigenetic blocks may co-opt REs or parts of their sequences as the new regulatory elements that take part in the control of gene expression, thus contributing to the emergence of new adaptive mechanisms [13]. Widespread functional domestication of the REs in the genomic site is the marker of its faster regulatory evolution and vice versa [14,15,16].

For the quantitative assessment of gene regulatory evolution, different types of regulatory sequences can be interrogated that can be assessed in different functional assays, mostly using the chromatin immunoprecipitation and sequencing (ChIP-seq) technology [17]. For example, these can be RE-linked transcription factor binding sites (TFBS) in the gene promoter or enhancer regions [9]. Alternatively, this can also be done for the histone modification tags that correlate with the structural chromatin alterations [18]. For example, at least five histone modification tags are frequently used to assess the functional state of the promoter. The presence of two histone modifications, H3K9me3 and H3K27me3, indicates transcriptionally silenced heterochromatin, while H3K4me3, H3K9ac, and H3K27ac correspond to open and transcriptionally active DNA, where “me” stands for methylation and “ac”—for acetylation [19].

For all of those types of regulatory elements, the RetroSpect method makes it possible to calculate relative and absolute metrics of RE-linked regulatory enrichment for both individual genes and the whole intracellular molecular pathways [13]. In RetroSpect, relative retroelement enrichment scores for individual genes are reflected by NGRE value (normalized gene RE-linked enrichment score). For the assessment of molecular pathways, NPII (normalized pathway involvement index) values are calculated, which reflect relative retroelement enrichment scores for the whole molecular pathways [13,19,20].

In the previous Retrospect applications [13,19,20], the most quickly evolving pathways and molecular processes were identified from the regulatory perspective in the human lineage, which were connected with adaptive immunity, RNA interference, sensory perception, sexual reproduction, lipids metabolism, detoxication of toxins, and other processes. In turn, the most slowly evolving processes from the gene regulation point of view dealt with ribosome biogenesis and protein biosynthesis, major transcriptional mechanisms, embryo development, and fundamental intracellular signaling pathways.

In this study, we combined Retrospect with the dN/dS approach to characterize in parallel the regulatory and structural evolutionary profiles for the genes involved in major human cancer pathways for the first time. Totally, 10,890 individual genes and 2972 molecular pathways were analyzed. Intriguingly, we observed a relatively low correlation between the structural and regulatory metrics at the gene level (~0.1), which appeared to be significantly higher at the level of molecular pathways (~0.4, *p* < 0.001). We deposited the full data on gene and pathway regulatory and structural evolutionary rates in the publicly available database called RetroSpect, accessible at https://retrospect.oncobox.com. We also analyzed the evolutionary dynamics of six cancer pathways of two major axes: Notch/WNT/Hedgehog and AKT/mTOR/EGFR. Most of them had a close to average both regulatory and structural components. However, the Hedgehog pathway had both components slower, whereas the Akt pathway had clearly accelerated structural evolution. In particular, the major hub nodes Akt and beta-catenin showed both components strongly decreased, whereas two major regulators of Akt TCL1 and CTMP had outstandingly high evolutionary rates. We also noticed structural conservation of serine/threonine kinases and the genes related to guanosine metabolism in cancer signaling: GPCRs, G proteins, and small regulatory GTPases (Src, Rac, Ras); however, this was compensated by the accelerated regulatory evolution.

## 2. Materials and Methods

### 2.1. dN/dS Data

The ratio of the number of nonsynonymous substitutions per non-synonymous site (dN) to the number of synonymous substitutions per synonymous site (dS) was used as structural evolutionary rate metrics for individual genes. Values of the dN/dS ratio for 11,534 individual human genes were extracted from the previous reports [21,22].

For each molecular pathway under investigation, the averaged value was calculated as the mean of dN/dS ratios for genes whose products take part in the respective pathway.

### 2.2. Retroelement Enrichment Data

Relative retroelement enrichment scores for genes (normalized gene RE-linked enrichment score, NGRE), which were previously calculated using ChIP-seq profiles of human cell lines, were extracted from our previous datasets [13,19,20] for six histone modifications (H3K4me1, H3K4me3, H3K9ac, H3K27ac, H3K27me3, and H3K9me3), and for transcription factor binding sites (TFBSs). NGRE reflects the ratio of RE-linked regulatory elements to all regulatory elements in the 10-kb frame centered around the gene canonical transcriptional start site [13,19,23].

NGRE values calculated using histone modification tags and using the occurrence of TFBS were available for five human cell lines (GM12878 for lymphoblastoid cells, Hela-S3 for cervical carcinoma, HepG2 for hepatocellular carcinoma, K562 for leukemia, MCF-7 for breast cancer) and for 13 cell lines (GM12878 and GM12891 for lymphoblast cells, Hela-S3, HepG2, K562, HEK293 and HEK293T for embryonal kidney, A549 for alveolar adenocarcinoma, SK-N-SH for neuroblastoma, HCT116 for colon carcinoma, Ishikawa for endometrial adenocarcinoma, MCF-10A for non-tumorigenic epithelium), respectively.

Relative retroelement enrichment scores for molecular pathways (normalized pathway involvement index, NPII) [13,19,20] were calculated in this study using an updated human molecular pathway database according to [24].

### 2.3. Functional Classification of Histone Modifications

H3K4me3, H3K9ac, and H3K27ac are typically considered as the tags of promoter/enhancer regions and active chromatin, and H3K27me3, H3K9me3—as the heterochromatin (transcriptionally silent chromatin) tags (Supplementary Table S1). H3K4me1 is specific both for poised and active enhancers or promotors, and its functional meaning depends on the distribution pattern. To simplify the calculation pipeline, H3K4me1 was considered in this paper as a tag of potentially active chromatin (Supplementary Table S1).

### 2.4. Analysis of Gene Ontologies

Gene ontology-enriched terms were defined for gene sets investigated using the *clusterProfiler* R package. *p*-values were adjusted accordingly to Benjamini and Hochberg’s false discovery rate (FDR) correction procedure. The statistical threshold for the FDR-adjusted *p*-value was 0.05.

### 2.5. Correlation Analysis

Spearman correlation coefficients and their *p*-values were calculated with the R *stats* package [25] and visualized using the R *corrplot* package [26]. We used a *p* < 0.05 threshold for the assessment of correlation significance.

### 2.6. Molecular Pathways Databank

Gene compositions of 3024 molecular pathways were taken from OncoboxPD collection [24] of uniformly processed and functionally annotated pathways from the following source databases: NCI Pathway interaction database [27], QIAGEN SABiosciences [28], Reactome [29], Biocarta [30], Kyoto Encyclopedia of Genes and Genomes (KEGG) [31], HumanCyc [32].

An architecture and gene composition of six cancer-related molecular pathways were extracted from the OncoboxPD collection [24].

### 2.7. Pathway Visualizations

Signaling pathways were visualized with RCy3 2.14.1 R package [33] and Cytoscape 3.9.0 [34]. The style was set to ‘Directed’ mode. We used continuous mapping for node size and color and discrete mapping for arrow color and node shape.

## 3. Results

### 3.1. Study Design

This study attempted to investigate in-depth the evolutional rates of human genes and molecular pathways, with specific attention to those connected with cancer progression, by simultaneously assessing both structural and regulatory alterations. To this end, we used the dN/dS ratio and relative retroelement enrichment score (RetroSpect method), respectively.

dN/dS is the ratio of nonsynonymous to synonymous substitutions, and the normalized retroelement enrichment score (NGRE) shows a proportion of regulatory elements of a gene that are held by REs, normalized to the relative content of REs in this gene vicinity. The regulatory elements were identified using Chip-Seq data for transcriptional factor binding sites (TFBS) and for DNA sites binding modified histones. The Chip-Seq data under analysis for various histone tags and transcription factor binding sites were established for 5–13 human cell lines depending on the nature of a functional site (histone and TFBS tags).

We then combined NGRE metrics for different functional tags to obtain a uniform retroelement enrichment regulatory score for every gene and pathway. Such NGRE-based metrics were then compared with the dN/dS metrics. To characterize molecular pathways, mean per pathway dN/dS ratio, and NGRE scores were calculated.

We then characterized in more detail six molecular pathways which belong to two main cancer-related axes: AKT-mTOR-EGFR cascade and Hedgehog-Notch-WNT signaling. AKT, mTOR, EGFR, Notch, WNT, and Hedgehog pathways also essentially contribute to the normal human embryo development, formation of organs and tissues, and regulation of basic cellular processes [35,36,37,38,39,40,41].

### 3.2. Aggregation of NGRE Scores

As defined in this study, NGRE is a relative enrichment of RE-linked regulatory elements in the 10-kb frame centered around the gene canonical transcriptional start site. NGRE values were calculated as a ratio of RE-linked to all regulatory elements using H3K4me1, H3K4me3, H3K9ac, H3K27ac, H3K27me3, H3K9me3 chromatin tags, and TFBS for 563 transcriptional factor proteins, established using ChIP-Seq data for different human cell lines (Table 1).

For every functional tag, we averaged the data for all cell lines under analysis to avoid possible effects of tissue-specific gene expression on the assessment of regulatory evolution rates. For example, NGRE _H3K9ac_ is the weighted average NGRE metric for GM12878, Hela-S3, HepG2, K562, and MCF-7 cell lines. Weights of cell line profiles were introduced to consider data quality. The profiles with smaller information gain (i.e., with a bigger proportion of “zero” values for individual genes) were tuned to contribute smaller to the final score. The weight of each cell line was calculated using the following formula: W*i* = (1 − z_i_)^3^, where z is the proportion of *zero* values in the *i* cell line profile (Supplementary Table S2). Thus, we obtained weighted average scores separately for each histone tag and for TFBS (NGRE_H3K27me3_, NGRE_H3K9me3_, NGRE_H3K9ac_, NGRE _H3K27ac_, NGRE _H3K4me3_, NGRE_H3K4me1,_ NGRE_TFBS_).

We then calculated the final aggregated NGRE metrics by combining the weighted average scores obtained for every functional tag according to the following algorithm:

- We first grouped the histone tag metrics according to their relation to active chromatin (H3K4me3, H3K9ac, H3K27ac, H3K4me1) or heterochromatin (H3K27me3, H3K9me3). Active chromatin NGRE_ac_ = (NGRE_H3K9ac_ + NGRE _H3K27ac_ + NGRE _H3K4me3_ + NGRE_H3K4me1_)/4; heterochromatin NGRE_hc_ = (NGRE_H3K27me3_ + NGRE_H3K9me3_)/2.
- Finally, using three functional tag components, we calculated aggregated NGRE metrics for the first time, which can be regarded as joint indexes of regulatory evolution for every gene under analysis (NGRE_AGG_):

NGRE_AGG_ = (NGRE_hc_ + NGRE_ac_ + NGRE_TFBS_)/3.

As a result, we obtained 11 metrics to investigate the evolution of genes independently of tissue type: dN/dS ratios, average NGRE based on separate histone tags (NGRE_H3K27me3_, NGRE_H3K9me3_, NGRE_H3K9ac_, NGRE _H3K27ac_, NGRE _H3K4me3_, NGRE_H3K4me1_), average NGRE based on active chromatin and heterochromatin (NGRE_ac_, NGRE_hc_), TFBS (NGRE_TFBS_), and final aggregated NGRE score (NGRE_AGG_), Supplementary Table S3.

### 3.3. Correlation between Evolutionary Rate Metrics for Individual Genes

We analyzed here a total of 10,890 genes that were common between the sets of genes with available dN/dS data (11,534 genes) and genes with ChIP-seq data (23,738–25,075 genes for different histone and TFBS tags). We observed statistically significant correlations between all types of NGRE-based metrics for the different functional tags and cell lines, thus underlining the congruent regulatory evolution of genes at different dimensions (different histone or TFBS tags). Correlation coefficients between NGRE values in different cell lines and tags were statistically supported (*p* < 0.001) and varied from 0.28 (NGRE_H3K4me3_ vs. NGRE_H3K9me3_ in MCF-7 cell line) till 0.952 (NGRE_TFBS_ in HEK293 vs. NGRE_TFBS_ in HEK293T cell line). Interestingly, higher correlations (0.754–0.901) were observed between NGRE scores for heterochromatin tags (H3K9me3 and H3K27me3) than between different active chromatin tags (0.58–0.89).

We then calculated rank correlations between dN/dS and NGRE metrics for the full list of genes under analysis (Supplementary Figure S1) and observed relatively low yet statistically significant correlations using all cell lines data (0.08–0.18, *p* < 0.001), Supplementary Figure S1.

We also considered separately a fraction of 710 genes included in six major cancer-related molecular pathways (AKT, mTOR, EGFR, and Notch, WNT, and Hedgehog pathways) that form two non-overlapping regulatory axes. In this subset, we observed the same correlation trend as described above for the full list of genes (Supplementary Figure S2).

To assess the significance level of NGRE, we tested if the NGRE distribution of a gene in different cell lines is significantly different (higher or lower) than the average level of NGRE among all genes in all cell line profiles. Totally, we found 379 genes where NGRE scores were distributed below the average value (*p*-adjusted < 0.05). Interestingly, 39 of them were involved in cancer-related molecular pathways (*ATXN2*, *AXIN1*, *BCL2L11*, *CAMK2B*, *CDH15*, *CDH17*, *CHUK*, *COL1A1*, *COL4A6*, *COL7A1*, *CSNK1G1*, *CTGF*, *CXCL12*, *CXCL6*, *FGF11*, *FGF5*, *FZD5*, *GABRA1*, *GNAS*, *GNRH2*, *HAPLN1*, *IHH*, *INSRR*, *ITGB2*, *LAMB2*, *LIFR*, *MAPK10*, *MUC1*, *PIK3R1*, *PPARGC1A*, *PSMA7*, *PTCH1*, *PTK7*, *SHC3*, *TCF4*, *TGFB2*, *TNFRSF14*, *TUBGCP2*, and *ZEB1*). At the same time, we did not detect genes with NGRE distribution above the average (*p*-adjusted > 0.95, Supplementary Table S4).

**Figure 1:**
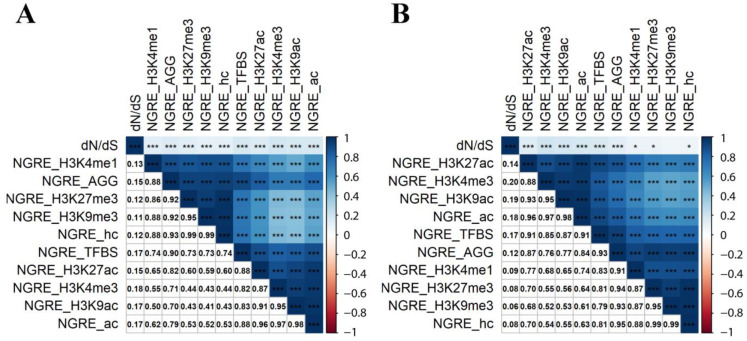
Rank pairwise correlation between 11 gene evolutionary rate metrics related to NGRE and dN/dS. (**A**) Correlations calculated using 10,890 gene profiles. (**B**) Correlations calculated using 710 gene profiles for six major cancer-related pathways. Upper matrix triangle and main diagonal are colored by rank correlation value, and asterisks denote *p*-value: * stands for *p* < 0.05 and *** for *p* < 0.001. Lower matrix triangle contains rank correlation coefficients in numeric format. The clustering method is Ward.D2 [43].

We then analyzed the correlation of dN/dS and NGRE metrics aggregated between all cell types, separately for all genes, and for 710 genes from the six above cancer-related pathways (Figure 1).

Again, we observed a low (0.08–0.206) but statistically significant correlation of dN/dS with NGRE metrics. Interestingly, correlated NGRE profiles formed two main clusters mostly related to (i) TFBS + active chromatin, and (ii) heterochromatin metrics (Figure 1); correlations within the clusters were (0.349–0.868) and (0.386–0.897), respectively.

### 3.4. Functional Groups of Genes with the Lowest and with the Highest Evolutionary Rate Ranks

Considering NGRE_AGG_ as the aggregated measure of the rate of regulatory evolution and dN/dS—of structural evolution, we identified in the whole gene set four major groups of genes, where: (1) gene regulatory evolution is accelerated, structural evolution is slow (334 genes); (2) regulatory evolution is slow, structural evolution is accelerated (298 genes); (3) both components are accelerated (547 genes); (4) both components are slow (561 genes). The criteria for such stratification were passing by the corresponding metrics of the thresholds of exceeding the top 0.8 quantile for accelerated and being lower than the bottom 0.2 quantile for slow gene evolution. Each of the above four groups was formed as the intersection of genes passing these inclusion criteria (Supplementary Table S5).

**Figure 2:**
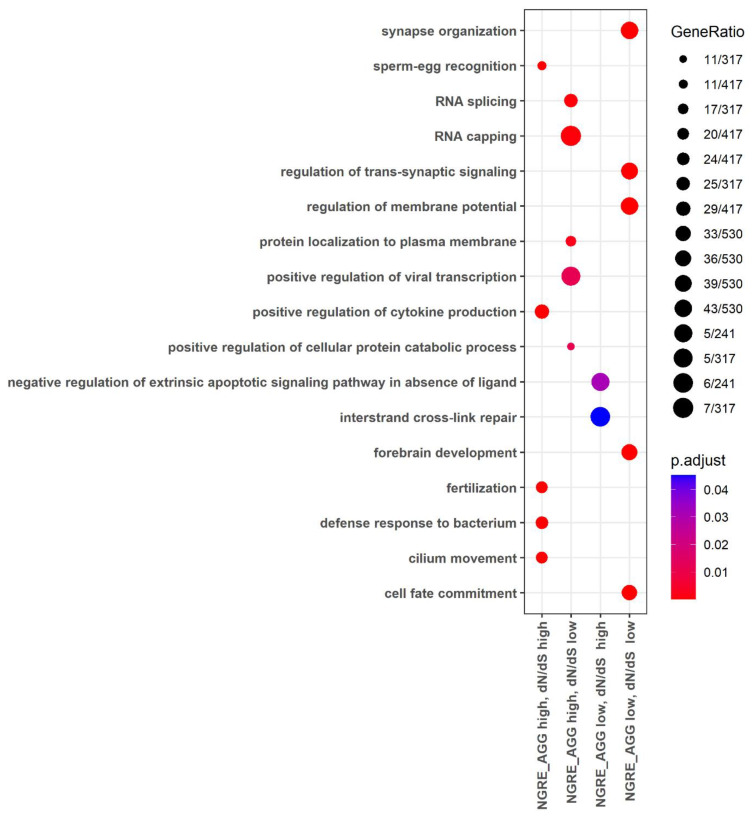
Gene Ontology terms enriched for the whole gene set in four groups of genes: (i) high both dN/dS and NGRE_AGG_ values, (ii) high NGRE_AGG_, low dN/dS values, (iii) low NGRE_AGG_, high dN/dS values, (iv) low both dN/dS and NGRE_AGG_ values.

The gene ontology (GO) analysis showed that the high rate of regulatory and structural evolution (high NGRE_AGG,_ high dN/dS) was observed for the processes related to immunity (e.g., activation of leukocytes and their subtypes, synthesis of cytokines, regulation of leukocyte cell-cell adhesion, regulation of lymphocyte proliferation), antibacterial response and restriction of the viral life cycle, sexual reproduction (spermatid differentiation, sperm-egg recognition, fertilization, sperm capacitation, germ cell development), cell movement (cilia- and microtubule-based movement, or flagellum-dependent cell motility) and others (Figure 2, Supplementary Table S6).

At the same time, the lowest rate of both structural and regulatory evolution was observed for the molecular processes regulating growth and development (e.g., forebrain development, neurogenesis and neuronal migration, heart and muscle tissue development), the basic processes of genetic information realization (e.g., transcription elongation from RNA polymerase II promoter) and maintaining DNA conformation (e.g., histone modification), basic mechanisms of export from cell and neurotransmitter secretion, learning and memory, some basic mechanisms of cellular motility, and processes of cation transport (Figure 2, Supplementary Table S6). In addition, there were common enriched processes here with the “high NGRE_AGG_, low dN/dS” group of genes: Wnt signaling, proteasomal protein catabolic process, protein polyubiquitination, transcription elongation from RNA polymerase II promoter, regulation of mRNA metabolic processes, RNA splicing (Supplementary Figure S3, Supplementary Table S6).

The other processes characteristics of the “high NGRE_AGG_, low dN/dS” group were as follows: mRNA processing, endocytic recycling, nuclear localization signal-bearing protein import into nucleus, regulation of viral transcription, intracellular transport of virus, and protein polyubiquitination (Figure 2, Supplementary Table S6).

Finally, in the “low NGRE_AGG_, high dN/dS” group, there were genes enriched in the GO processes of regulation of extrinsic apoptotic signaling pathway in the absence of ligand, negative regulation of signal transduction in the absence of ligand, and interstrand cross-link repair (Figure 2, Supplementary Table S6).

In the same way, we also assessed such four groups for the subset of 710 cancer pathway genes: (1) NGRE_AGG_ high, dN/dS low (21 genes); (2) NGRE_AGG_ low, dN/dS high (18 genes); (3) double high (39 genes); (4) double low (27 genes), Supplementary Table S7.

In contrast to results for the whole gene set, common GO terms were found for all four groups derived from the 710 genes set due to their initial attribution to six major cancer-related pathways (Supplementary Figure S4). Such common GO terms (marked as “duplicated” Supplementary Table S8) were not considered further to avoid false positive results. Instead, we focused on the group-specific GO terms.

**Figure 3:**
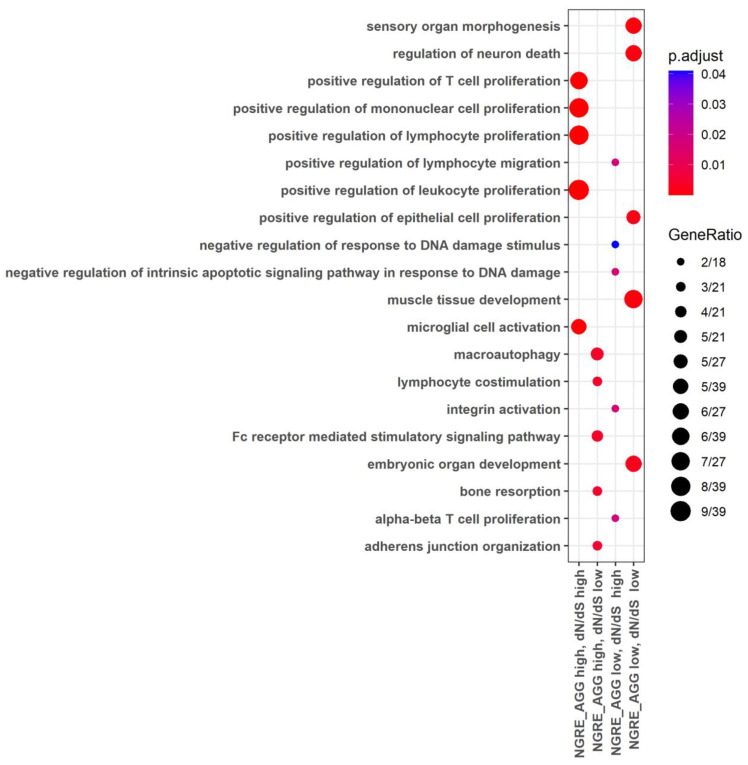
Gene ontology terms enriched for cancer pathway-related limited 710-gene set in four groups of genes: (i) high both dN/dS and NGRE_AGG_ values, (ii) high NGRE_AGG_, low dN/dS values, (iii) low NGRE_AGG_, high dN/dS values, (iv) low both dN/dS and NGRE_AGG_ values.

The GO analysis for the “double high” group of cancer pathway genes showed that they were enriched in the processes dealing with immunity (e.g., proliferation, differentiation, activation, migration of immune cells, and synthesis of cytokines), JAK-STAT signaling, antibacterial response, temperature homeostasis, calcium transport, cell adhesion, and other processes (Figure 3, Supplementary Table S8).

At the same time, for the “double low” group, we observed the fundamental processes of micro RNA transcription and RNA stability, trans-synaptic signaling, response to hypoxia, growth, and development (e.g., neuronal differentiation, angiogenesis, and embryo development), Figure 3, Supplementary Table S8.

In the “high NGRE_AGG_, low dN/dS” group, we identified some processes related to immunity (Fc receptor-mediated stimulatory signaling pathway, T-cell costimulation, lymphocyte costimulation, immune response-regulating cell surface receptor signaling pathway involved in phagocytosis), macroautophagy, bone matrix processes, development of organs and skeletal system, and glucose homeostasis (Figure 3, Supplementary Table S8).

The “low NGRE_AGG_, high dN/dS” group had genes enriched in terms of activation of integrins, negative regulation of intrinsic apoptotic signaling pathway in response to DNA damage, T-cell proliferation and migration, and production of chemokines (Figure 3, Supplementary Table S8).

### 3.5. Molecular Pathways with the Lowest and with the Highest Evolutionary Rate Ranks

In parallel, we analyzed the structural and regulatory evolution of the human molecular pathways. In this case, Normalized Pathway Involvement Index (NPII) scores were calculated for measuring the regulatory evolution of pathways, similar to the NGRE scores for the gene-wise level (Supplementary Table S3). Also, for every pathway, we calculated the dN/dS ratio as the average value of that for all genes included in this pathway; this was used as a metric of the structural evolution of the pathways. Both types of data could be calculated for 2972 out of a total of 3024 molecular pathways (Supplementary Table S3).

Based on the NPII values, we assessed rank correlations between the regulatory metrics of evolutionary rate in different sample types (Supplementary Figure S5). As before, we observed different clustering for the active chromatin and for the heterochromatin NGRE values. A strong correlation (0.83–0.98, *p* < 0.001) was observed between all cell lines for the TFBS-based NPII, except for the single cell line MCF-10A. Correlation in the active chromatin and heterochromatin clusters varied between 0.6 and 0.9 and between 0.78 and 0.96, respectively.

Overall, the TFBS-based NPII profiles showed higher correlation with both active chromatin and heterochromatin marks (0.65–0.83 and 0.62–0.89, respectively) than active chromatin and heterochromatin profiles with each other (0.42–0.72). Aggregated profiles for the NPII_ac_, NPII_hc_, and NPII_TFBS_ were averaged separately. Overall, NPII_AGG_ values were obtained similarly to NGRE_AGG_. This is an averaged profile consisting of three components: the average NPII scores for the active chromatin, heterochromatin, and TFBS with weight coefficients of 1/3 for each component. The weights of the individual cell lines were defined as described for the gene level.

**Figure 4:**
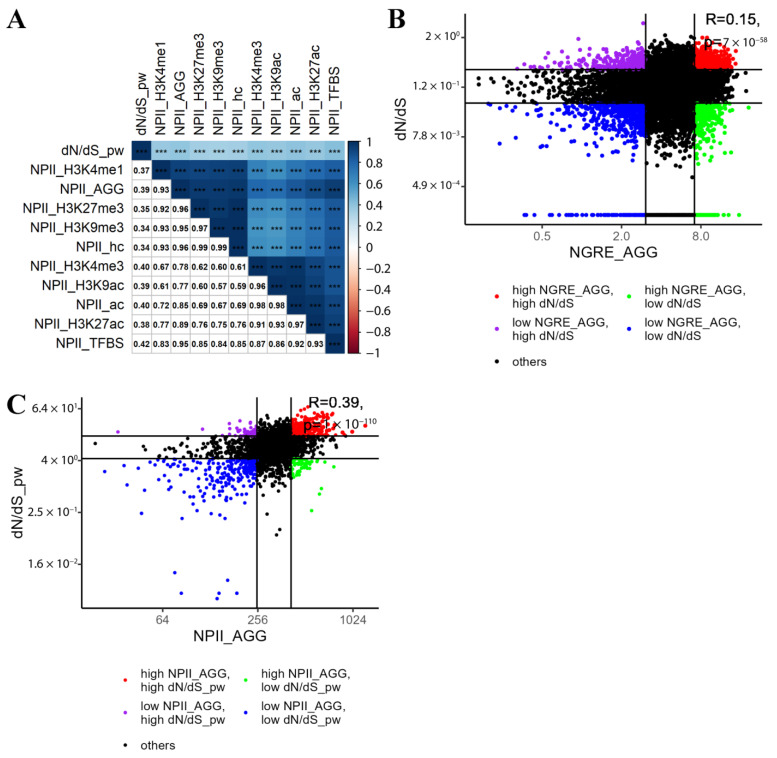
Rank pairwise correlation between 11 gene evolutionary rate metrics related to NPII and pathway dN/dS. (**A**) upper matrix triangle and main diagonal are colored by rank correlation value, and asterisks denote *p*-value: *** for *p* < 0.001. Lower matrix triangle contains rank correlation coefficients in numeric format. The clustering method is Ward.D2 [43] (**B**) rank correlation between dN/dS and the aggregated NGRE metric for individual genes. Top and bottom 0.2 quantiles are colored to show groups with high and/or low evolutionary metrics, accordingly. (**C**) rank correlation between dN/dS and the aggregated NPII metric for molecular pathways. Top and bottom 0.2 quantiles are colored to show groups with high and/or low evolutionary metrics accordingly.

The analysis of 2972 molecular pathways showed a positive correlation between mean dN/dS per pathway and NPII: from 0.34 to 0.42 (Figure 4), which was statistically significant and strongly exceeded the ~0.1 correlation seen at the gene level (Figure 4B). The highest correlation with dN/dS was detected for the NPII_TFBS_ (0.42, *p* < 0.001).

Similarly to the previous gene-wise level analysis, depending on NPII_AGG_ and pathway dN/dS (dN/dS_PW_) values and using the same thresholds, four groups of molecular pathways were identified (Supplementary Table S9): (i) with high NPII_AGG_ and low dN/dS_PW_ (66 pathways); (ii) with low NPII_AGG_ and high dN/dS_PW_ (47 pathways); (iii) with high both NPII_AGG_ and dN/dS_PW_ (249 pathways); (iv) with low both NPII_AGG_ and dN/dS_PW_ (242 pathways). Each of the above four groups was formed as the intersection of pathways passing these inclusion criteria (Supplementary Table S9).

**Figure 5:**
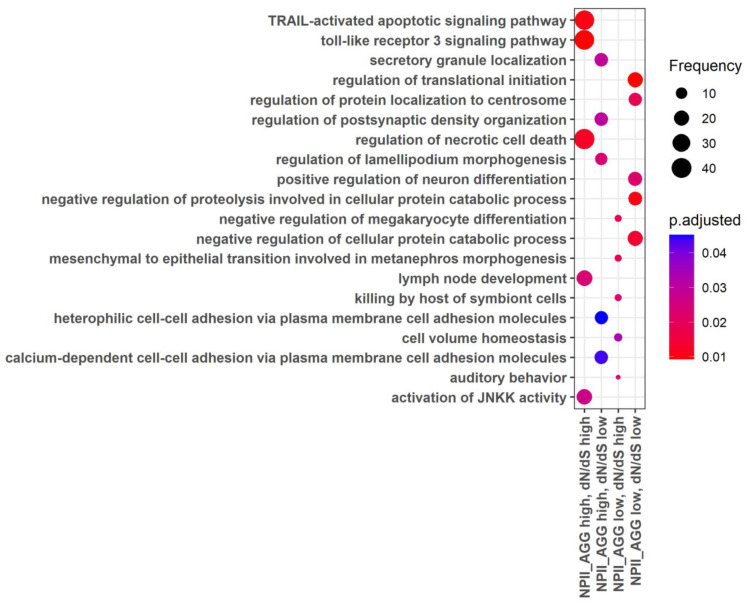
Top 5 GO tags for genes included in the four groups of pathways: with high NPII_AGG_ and low dN/dS_PW_ (66 pathways); low NPII_AGG_ and high dN/dS_PW_ (47 pathways); high NPII_AGG_ and high dN/dS_PW_ (249 pathways); low NPII_AGG_ and low dN/dS_PW_ (242 pathways). The threshold for high/low values is top/bottom 20% of pathways sorted by a corresponding value.

Each pathway was functionally annotated with GO terms as described in [24]. To this end, the pathway gene lists underwent enrichment analysis, and all significantly enriched GO terms were assigned to the pathway as specific tags. Thus, each pathway obtained few GO terms. Some of such terms were common for several pathways (Supplementary Figure S6). The top five of the most frequently occurring GO terms for each functional pathway group are shown in Figure 5. Full lists of GO terms are given in Supplementary Table S10. For the above four groups, there were a total of 60, 30, 305, and 513 unique GO terms, respectively.

For double high set of pathways, the relevant biological processes include apoptotic and necrotic cell death (e.g., TRAIL-activated apoptotic signaling, regulation of programmed necrotic cell death, regulation of necroptotic process), membrane transport and ion homeostasis (e.g., regulation of chloride transport, cellular anion homeostasis, cellular sodium ion homeostasis, phosphate ion homeostasis, negative regulation of calcium ion import, phosphate ion transmembrane transport, zinc ion transmembrane transport, negative regulation of potassium ion transmembrane transporter activity, pyrimidine-containing compound transmembrane transport, basic amino acid transmembrane transport, azole transmembrane transport, negative regulation of membrane potential, proline transmembrane transport, nucleotide transmembrane transport), toll-like receptor 3 signaling, immunity (e.g., lymph node development, regulation of interleukin-6-mediated signaling pathway, activation of macrophages), activation of JNKK activity, positive regulation of cellular extravasation, mitosis (mitotic centrosome separation, attachment of mitotic spindle microtubules to kinetochore, metaphase plate congression, regulation of mitotic cell cycle spindle assembly checkpoint, DNA replication checkpoint), myelinization, dendritic cell cytokine production, and the others (Figure 5, Supplementary Table S10).

The double low pathway set appeared to be connected with the following: transcription and translation (regulation of translational initiation, regulation of RNA binding, rRNA metabolic process, rRNA processing, maturation of SSU-rRNA, negative regulation of RNA splicing, mRNA destabilization, maturation of LSU-rRNA, positive regulation of transcription by RNA polymerase III, regulation of mRNA processing, mRNA cis splicing, via spliceosome, mRNA polyadenylation, mRNA splice site selection, RNA polyadenylation), DNA conformation (regulation of chromatin silencing, protein-DNA complex disassembly, DNA methylation-dependent heterochromatin assembly, DNA integration, histone H4 deacetylation, heterochromatin organization), neural development (positive regulation of neuron differentiation, neural tube patterning, embryonic eye morphogenesis, corpus callosum development, cranial nerve development, embryonic brain development, cerebellar Purkinje cell layer morphogenesis), and the others (Figure 5, Supplementary Table S10).

For the high dN/dS_PW_ and low NPII_AGG_ pathways, the relevant processes included water homeostasis at cell and organism levels (cellular hyperosmotic response, cellular response to salt stress, cell volume homeostasis, mineralocorticoid metabolic process, aldosterone biosynthetic process), kidney development (nephron tubule formation, metanephric tubule, and epithelium development), killing by host of symbiont cells, auditory behavior, negative regulation of megakaryocyte differentiation, base-excision repair, catecholamine uptake involved in synaptic transmission, glucocorticoid biosynthetic process, inositol metabolic process, and the others (Figure 5, Supplementary Table S10).

Low dN/dS_PW_ and high NPII_AGG_ pathways were associated with calcium-dependent cell-cell adhesion via plasma membrane adhesion molecules, dense core granule transport, localization and regulation of postsynaptic density organization, regulation of lamellipodium morphogenesis, response to acidic pH, the establishment of T-cell polarity, membrane raft organization, mitochondrial calcium ion homeostasis, amyloid precursor protein biosynthetic process, endoplasmic reticulum(ER)-related processes (regulation of vesicle size, regulation of ER to Golgi vesicle-mediated transport, maintenance of protein localization in ER), and the others (Figure 5, Supplementary Table S10).

Then, we applied stronger thresholds for the top/bottom pathways: 1%, 5%, and 10%, in addition to the used threshold of top/bottom 20%, and annotated the obtained groups of pathways (Table 2) with the gene ontology terms (Supplementary Figures S7–S9, Supplementary Tables S11–S13).

The top of GO terms was stable for groups with the same metrics (e.g., low dN/dS and high NPII_AGG_) that include more than 30 pathways. Then, it gradually changed with an increase of the threshold. For the strongest threshold, the group of 20 pathways with both low dN/dS and NPII_AGG_ was linked with carbohydrate metabolic process, intrinsic apoptotic signaling, regulation of cell-cell adhesion, activation of protein kinase B activity, and others (Supplementary Table S13, Supplementary Figure S9). Pathway set with both high dN/dS and NPII_AGG_ is characterized by amino acid metabolic processes, cell differentiation involved in embryonic placenta development, interleukin-8 production, macrophage differentiation, necrotic cell death, and others (Supplementary Table S13, Supplementary Figure S9). The other two groups contain one pathway each (Table 2). Scavenging by Class B Receptors pathway is responsible for the binding of chemically modified lipoproteins, and the branch of regulation of nuclear SMAD2 3 signaling pathway negatively regulates cell growth (Supplementary Table S13, Supplementary Figure S9).

### 3.6. Database of Human Genes Structural and Regulatory Evolutionary Rate

Using the above NGRE, NPII, dN/dS, and dN/dS_pw_ data, we created a public database termed RetroSpect-DB cataloging rates of structural and regulatory evolution of 10,890 human genes and 2972 molecular pathways. The database is available at https://retrospect.oncobox.com.

### 3.7. Major Cancer-Related Pathways in the Context of Structural and Regulatory Evolution of Human Genes

#### 3.7.1. Rank of Cancer Pathways by Evolutional Metrics

We assessed NPII_AGG_ and dN/dS_PW_ levels of six major cancer-related pathways which form two strongly connected gene networks: (i) pathways of AKT, mTOR, and EGFR signaling, and (ii) Notch, WNT, and Hedgehog pathways relative to the whole set of 2972 molecular pathways under study. For both structural and regulatory evolution metrics, these six cancer pathways showed average scores in an overall distribution (Supplementary Figure S10). Thus, six main cancer-related pathways show roughly median rates of both structural and regulatory evolution in humans. The major bias was seen for the Hedgehog pathway, which showed the lowest rates of both structural and regulatory evolution (Supplementary Figure S10).

A similar tendency was also seen on the level of individual genes forming these six cancer pathways (Supplementary Figure S11).

#### 3.7.2. Evolution of Functional Nodes of Cancer Pathways

**Figure 6:**
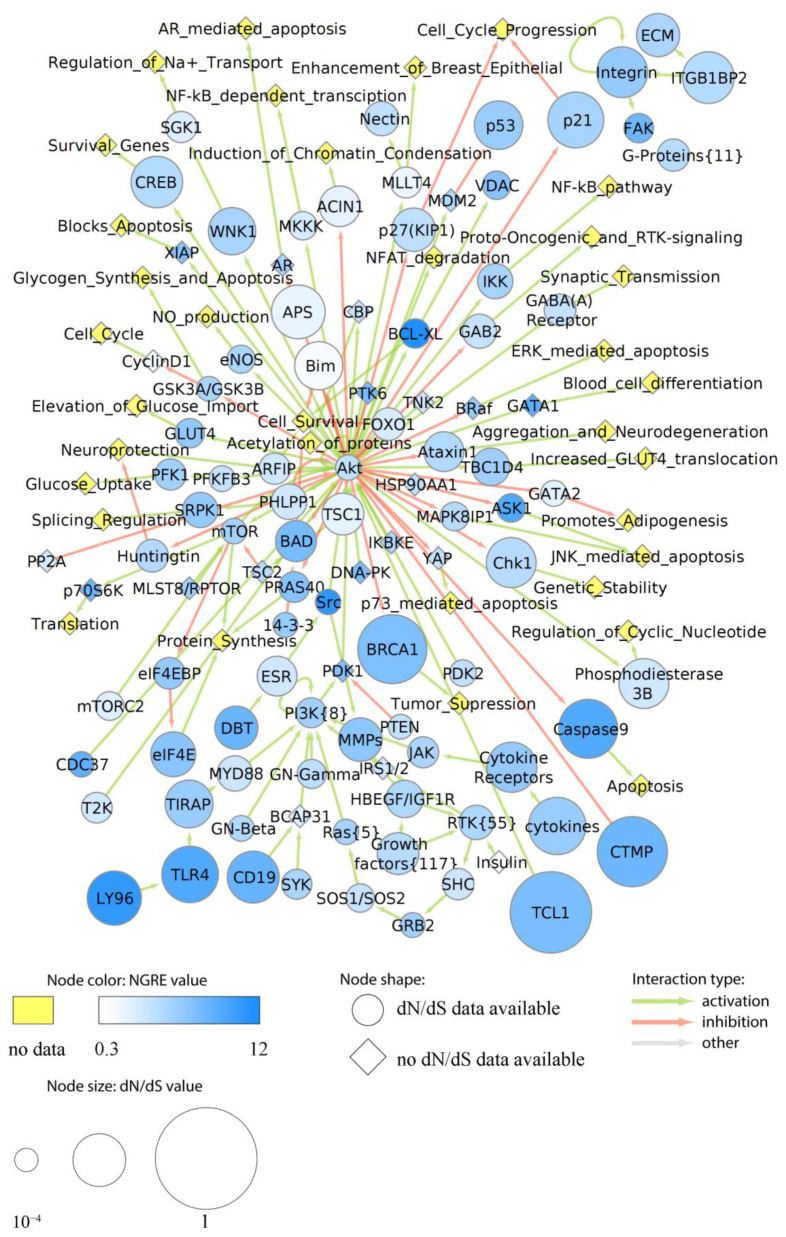
Evolutionary chart of human molecular pathway Akt. Blue color intensity reflects NGRE_AGG_ value, size of nodes is proportionate to averaged dN/dS score. Nodes where dN/dS information is missing are given in rhombic shape. Nodes where no NGRE_AGG_ values are available are shown in yellow. A number in brackets stands if the node has the same name but a different number of molecular participants in different pathways under analysis.

**Figure 7:**
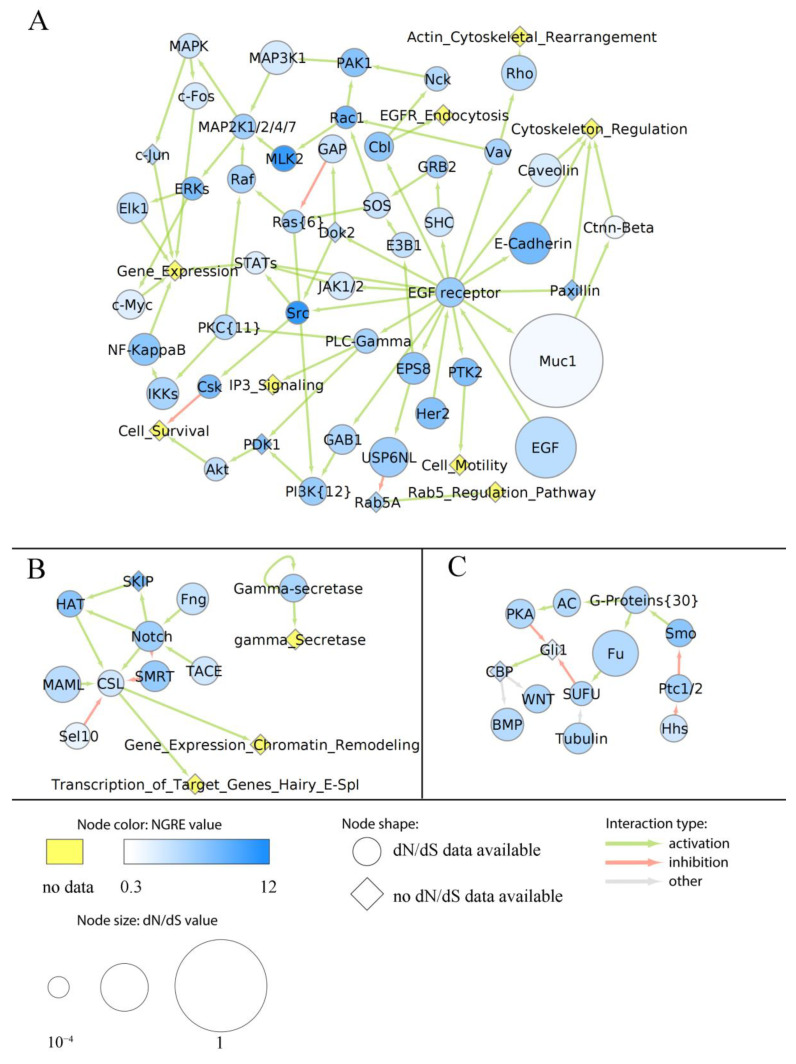
Evolutionary chart of human molecular pathway of EGFR signaling (**A**), Notch **(B**), and Hedgehog (**C**) pathways. Blue color intensity reflects NGRE_AGG_ value, size of nodes is proportionate to averaged dN/dS score. Nodes where dN/dS information is missing are given in rhombic shape. Nodes where no NGRE_AGG_ values are available are shown in yellow. A number in brackets stands if the node has the same name but a different number of molecular participants in different pathways under analysis.

**Figure 8:**
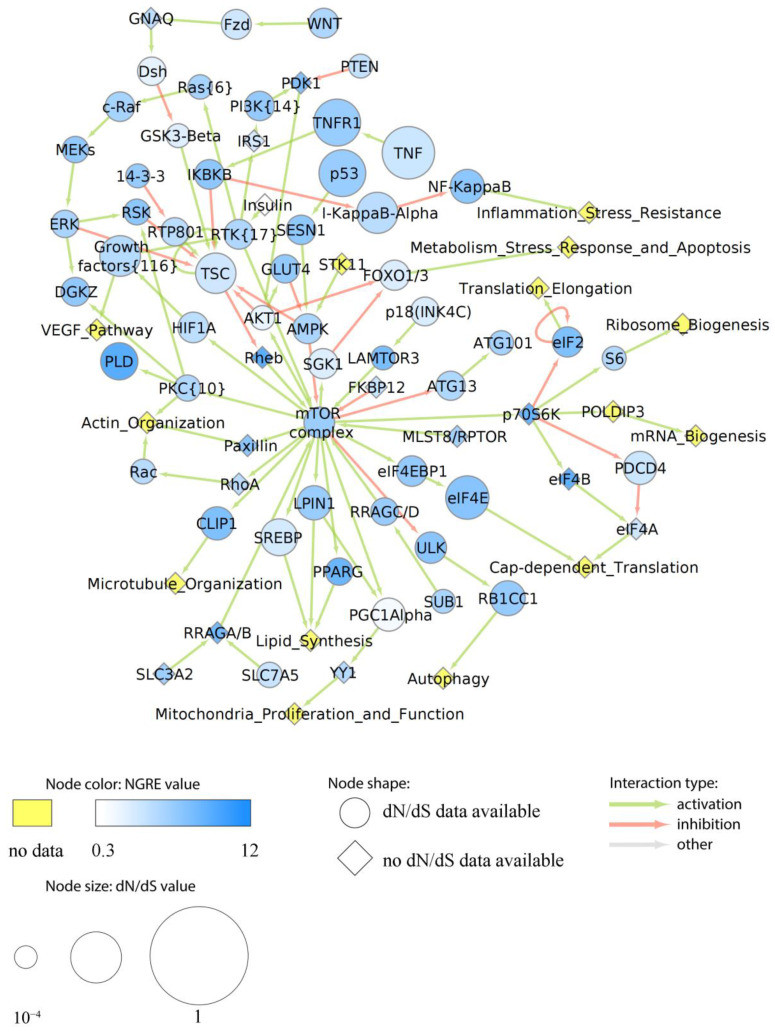
Evolutionary chart of human molecular pathway of mTOR signaling. Blue color intensity reflects NGRE_AGG_ value, size of nodes is proportionate to averaged dN/dS score. Nodes where dN/dS information is missing are given in rhombic shape. Nodes where no NGRE_AGG_ values are available are shown in yellow. A number in brackets stands if the node has the same name but a different number of molecular participants in different pathways under analysis.

**Figure 9:**
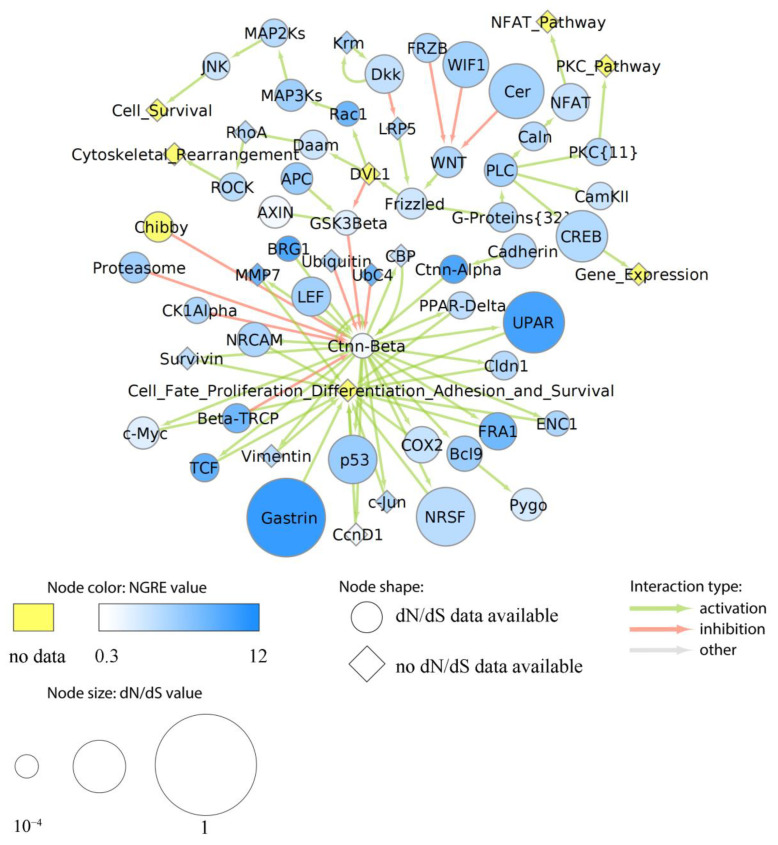
Evolutionary chart of human molecular pathway of WNT signaling. Blue color intensity reflects NGRE_AGG_ value, size of nodes is proportionate to averaged dN/dS score. Where dN/dS information is missing, a node is given in rhombic shape. Nodes where no NGRE_AGG_ values are available are shown in yellow. A number in brackets stands if the node has the same name but a different number of molecular participants in different pathways under analysis.

More specifically, we assigned dN/dS values to each node of the above six cancer pathways as the averaged dN/dS ratios of all genes included in the respective node. Similarly, aggregated NGRE values were calculated for each node. In six pathways under study, we found no statistically significant correlation between NGRE_AGG_ and dN/dS scores at the level of pathway nodes (Supplementary Table S14). The lack of correlation here may suggest alternative ways of the evolution of nodes in the pathways under study. We then visualized specific pathway profiles of averaged dN/dS and NGRE_AGG_ scores to assess the evolutional rates of each individual node (Figure 6, Figure 7, Figure 8, Figure 9 and Supplementary Figure S12).

The Akt pathway showed an average level for the regulatory evolution metric NPII_AGG_ and a position above average for the structural evolution metric dN/dS_PW_ (Supplementary Figure S10). We found that in the Akt pathway, TCL1 (TCL1 family AKT coactivators A, B), CTMP (negative regulator of Akt), BRCA1 (major component of a complex that repairs DNA double-strand breaks), TLR4 and LY96 (toll-like receptor 4 and its associated protein), CD19 (B-lymphocyte surface antigen B4), and Caspase9 (initiator caspase of the apoptotic pathway) nodes showed high both dN/dS and NGRE_AGG_ values, which suggests that they have simultaneously high rates of structural and regulatory evolution (Figure 6).

Bim (apoptotic activator) and APS (antiphospholipid syndrome related) nodes showed relatively high rates of structural evolution and very low rate of regulatory evolution, whereas BCL-XL (apoptosis inhibitor) node showed a medium rate of the structural and high rate of regulatory evolution. Also, we found that the regulatory evolution component strongly predominates for the following protein kinases: Src, ASK1, and FAK. The majority of nodes had balanced nearly average levels of dN/dS and NGRE_AGG_ values (Figure 6). Interestingly, the Akt node itself, which is the major hub of the whole related molecular network (Figure 6), had remarkably low levels of both dN/dS and NGRE_AGG,_ which suggests its very high degree of evolutionary conservation.

In the EGFR pathway (overall average ranking by each of NPII_AGG_ and dN/dS_PW_ scores), we observed relatively low structural (dN/dS) and average regulatory (NGRE_AGG_) evolution rates for the central hub gene of the epidermal growth factor (EGF) receptor, EGFR (Figure 7A). In contrast, its major interactor—the EGF node—showed high dN/dS and mostly average NGRE_AGG_ values. We observed the opposite figure of high NGRE_AGG_ and average dN/dS values for EGFR co-receptor HER2, for transcriptional factor NF-KappaB, focal adhesion kinase (FAK) that is a major regulator of cell motility, and E-Cadherin node that mediates epithelial cell junctions (Figure 7). Interestingly, small regulatory GTPases Src, Ras, and Rac1 all had high NGRE_AGG_ and low dN/dS, thus clearly suggesting a regulatory trend prevailing in their molecular evolution. The same pattern was observed for the non-receptor tyrosine and serine/threonine kinases: C-terminal Src kinase (Csk) and mixed lineage kinase 2 (MLK2), extracellular signal-regulated kinases node (ERKs), which includes MAPK1 and MAPK3. In contrast, glycoprotein mucin 1 (Muc1) node had very high dN/dS and low NGRE_AGG_. In cancer, Muc1 can form an extended hydrophilic interface on the surface of cell membranes that prevents cancer cell recognition by the immune cells and can also inhibit penetration of hydrophobic chemotherapeutics [44]. It also has a role in the epithelial–mesenchymal transition during carcinogenesis [45].

In the Notch signaling pathway (overall average NPII_AGG_ and dN/dS_PW_), the core Notch node and the nodes HAT and SMRT showed average or slightly increased NGRE_AGG_ (Figure 7B). SKIP, a coactivator that enhances transcription from some RNA polymerase II promoters, had a relatively high regulatory evolutionary rate. At the same time, all nodes had average dN/dS values with small variations (Figure 7B).

In the Hedgehog pathway (overall, both NPII_AGG_ and dN/dS_PW_ scores were below average), the nodes showed relatively slow structural and regulatory evolutionary rates, including the core node Hhs (sonic hedgehog), Figure 7C. The node for G-protein coupled receptor (GPCR) Smo showed the average NGRE_AGG_ value but a low dN/dS level. In turn, node Fu (for positive regulator of transcriptional factor Gli) showed a relatively high rate of structural evolution but modest NGRE_AGG_ (Figure 7C).

In the mTOR pathway (overall average NPII_AGG_ and dN/dS_PW_), all nodes showed comparable average rates of structural evolution (Figure 8). However, phospholipase D (PLD), Eukaryotic translation initiation factor 4B (eIF4B), Ras homolog Rheb, ribosomal protein S6 kinase p70S6K and peroxisome proliferator-activated receptor gamma (PPARG) nodes showed a relatively high NGRE_AGG_ score which indicates accelerated regulatory evolution. NF-kappaB node (see above) also showed relatively high NGRE_AGG_. The others, including the core pathway node mTOR complex, had roughly balanced average levels of both dN/dS and NGRE_AGG_ scores.

Finally, in the WNT pathway (overall average NPII_AGG_ and dN/dS_PW_), most of the nodes showed decreased dN/dS values, including the core node Wnt (Figure 9). However, the nodes for Gastrin and UPAR (urokinase plasminogen activator surface receptor) showed enhanced both dN/dS and NGRE_AGG_ values. We also detected relatively high values of NGRE_AGG_ for the following nodes: ATP-dependent chromatin remodeler BRG1; alpha-catenin Ctnn-Alpha; Beta-TRCP, a checkpoint protein that inhibits CDK1 activity in response to genotoxic stress by mediating the degradation of CDC25A; transcription factors TCF and FRA1; ubiquitin-conjugating enzyme UbC4; matrix metalloprotease MMP7; and Rac1. AXIN node, which functions as a negative regulator of this pathway, showed low NGRE_AGG_ but almost average dN/dS value. Finally, in the major hub node of this pathway, Ctnn-Beta for beta-catenin protein, we observed outstandingly low both NGRE_AGG_ and dN/dS values.

## 4. Discussion

For the first time, we simultaneously investigated the whole-genome and whole-interactome levels of the aggregated metrics of the regulatory and structural evolution of individual genes and molecular pathways. For the regulatory evolution, such metric was built based on the profiles of 563 transcriptional factor-binding sites and of genomic distribution profiles of six specific histone modifications, each for 5–13 human cell lines. As the measure of the structural evolution of genes, we used the dN/dS ratio, which is the proportion of non-synonymous to synonymous mutations in protein-coding genes and, therefore, reflects the rate of structural changes of amino acid sequences in a protein [46,47].

Interestingly, the aggregated gene-wise scores of regulatory evolution (NGRE_AGG_) showed a very low yet statistically significant correlation with the dN/dS values of individual genes (rank correlation ~0.1, *p* < 0.001), whereas at the level of the molecular pathway-wise metrics (NPII_AGG_ for regulatory evolution), this correlation was significantly higher (~0.4, *p* < 0.001), Figure 1 and Figure 4.

For the first time, we could uncover structural and regulatory evolutionary rates for human genes and their groups and for the entire molecular pathways. For the whole gene set (10,890 genes), we selected four major groups where: (1) regulatory evolution is accelerated, structural evolution is slow (334 genes); (2) regulatory evolution is slow, structural evolution is accelerated (298 genes); (3) both regulatory and structural evolution is accelerated (547 genes); (4) both regulatory and structural evolution is slow (561 genes). Similarly, we identified the respective four groups for the set of 2.972 molecular pathways with 66, 47, 249, and 242 members, accordingly.

The gene ontology analysis showed that the high rate of both regulatory and structural evolution was characteristic of the genes and pathways related to immunity and antibacterial response. At the same time, the slowest rate of both components was seen for the developmental processes and their regulation, and for the basic processes of realization of genetic information and for structural organization of DNA. In addition, many specific processes were connected with the combinations of (1) fast structural and slow regulatory and (1) slow structural and fast regulatory evolution (Figure 2 and Figure 5, Supplementary Tables S6 and S10).

Some biological processes simultaneously relate to the groups that may have both low and high evolutionary metrics, e.g., in the case of relevant GO terms for molecular pathways (Figures S3, S4 and S6, Supplementary Tables S6, S8 and S10). It could be explained by the opposite evolutionary trends in different subsets of genes involved in such processes.

To summarize, we created, to our knowledge, the first comprehensive database in RetroSpect with the regulatory and structural evolutionary rates of 10,890 human genes and 2972 of their functional groups (molecular pathways). The database is publicly available at https://retrospect.oncobox.com.

In this study, we analyzed in detail the evolutionary dynamics of two major cancer axes: Notch/WNT/Hedgehog (Figure 7B,C, Figure 9 and Supplementary Figure S12) and AKT/mTOR/EGFR (Figure 6, Figure 7A, Figure 8 and Supplementary Figure S12). These pathways contain several nodes with high levels of both structural and regulatory evolution components (top 20% of all pathway nodes sorted by dN/dS ratio and NGRE_AGG_): UPAR, Gastrin, CD19, TLR4, CTMP, Caspase 9, and LY96. In addition, the PLD node has the highest level of NGRE_AGG_ and the average value of dN/dS score. The nodes with high NGRE_AGG_ but low dN/dS (top 20% and bottom 20%, respectively) were: CDC37, Src, BRG1, and Rac1. The opposite figure (bottom 20% by NGRE_AGG_, top 20% by dN/dS) was seen for the Muc1 and APS nodes. Akt, Ctnn-Beta, GSK3-Beta, and STATs were the nodes that had the slow rates by both components (bottom 20% by both dN/dS and NGRE_AGG_).

In addition to the combination of “top” or “bottom” genes, we found that the structural evolution component strongly predominates for Bim, PGC1Alpha, EGF, TSC1, TNF, ACIN1, Phosphodiesterase 3B, NRSF, AXIN, TCL1, ITGB1BP2, p21, and CREB nodes according to the NGRE_AGG_ to dN/dS ratio (Supplementary Table S15). In turn, regulatory evolution component strongly prevails for LAMTOR3, 14-3-3, GRB2, SUB1, GSK3A/GSK3B, S6, PTEN, STATs, GSK3-Beta, Ctnn-Beta, and ATG101 nodes (Supplementary Table S15).

Overall, we found that most of the six human cancer core pathways investigated here in detail have close to average regulatory and structural evolution rates. However, the Hedgehog pathway showed slower both regulatory and structural evolution, whereas the Akt pathway had clearly accelerated structural evolution. In most of the nodes, both components were balanced and close to the average values. The major cancer hub nodes Akt and beta-catenin showed strongly decreased evolutionary rates for both components. This poses the question of whether there exists a linkage between the evolutionary rates and numbers of proteins interconnected by the hub node, which has to be investigated in the future.

Interestingly, the two major regulators of Akt: TCL1 (TCL1 family AKT coactivators A, B) and CTMP (negative regulator of Akt), in contrast, showed outstandingly high evolutionary components for both. For another important cancer pathwayhub node MTOR—both components were on the average level, and for the hub EGFR—slightly decreased structural but average regulatory components.Furthermore, we noticed structural conservation of the genes related to guanosine metabolism in cancer signaling: GPCRs, and small regulatory GTPases (Src, Rac, Ras). Interestingly, in all of them, this was compensated by the accelerated regulatory evolution components.

We hope that this study and the RetroSpect database will be of interest to those working in the fields of cancer pathway analysis and human molecular evolution.

## 5. Conclusions

Structural and functional components make it possible to characterize the molecular evolution of human genes and of the whole molecular pathways in depth. Here we cataloged human genes and molecular pathways in relation to their structural and regulatory evolution rates. For the first time, we combined the dN/dS analysis (a metric of structural evolution of genes) with the new metric of regulatory evolutionary rates. Overall, we observed weak (~0.1, *p* < 0.001) yet statistically significant correlation between the metrics of structural and regulatory evolution for single genes and a stronger (~0.4, *p* < 0.001) correlation for the level of molecular pathways, which may suggest coordinated reshaping of their components during evolution.

Special attention was made to six major cancer-related molecular pathways, which were investigated in detail. Interestingly, we observed greater structural and regulatory conservation for the major hub genes participating in cancer pathways and having an overall bigger number of interactions. In contrast, gene products located on the pathway periphery and having fewer interactions showed greater rates of both structural and regulatory evolution.

## Figures and Tables

**Table 1:** Types of data for NGRE scoring (histone and TFBS tags) and the corresponding cell lines.

Functional Tag	Cell Line
*Active Chromatin:* H3K4me1, H3K4me3, H3K9ac, H3K27ac; *Heterochromatin*: H3K27me3, H3K9me3	GM12878—lymphoblastoid cells, Hela-S3—cervical carcinoma cells, HepG2—hepatocellular carcinoma cells, K562—leukemia cells, MCF-7—breast cancer cells
Transcriptional Factor Binding Sites (TFBS) for 563 transcriptional factor proteins	GM12878—lymphoblastoid cells, Hela-S3—cervical carcinoma cells, HepG2—hepatocellular carcinoma cells, K562—leukemia cells, MCF-7—breast cancer cells, HEK293—immortalized embryonal kidney cells, HEK293T—daughter cell line that was derived from HEK293 by transfecting with a plasmid expressing a temperature-sensitive version of the SV40 large T antigen [42] A549—alveolar adenocarcinoma cells, SK-N-SH—neuroblastoma cells, HCT116—colon carcinoma cells, Ishikawa—endometrial adenocarcinoma cells, MCF-10A—non-tumorigenic epithelial cells, GM12891—lymphoblastoid cells

**Table 2:** Size of an intersection of top/bottom pathways sorted by NPII_AGG_ and dN/dS.

Threshold for Top or Bottom	Low dN/dS, Low NPII_AGG_	High dN/dS, High NPII_AGG_	High dN/dS, Low NPII_AGG_	Low dN/dS, High NPII_AGG_
20%	242	249	47	66
10%	154	158	12	34
5%	85	83	2	17
1%	20	19	1 (Scavenging by Class B Receptors pathway)	1 (Regulation of nuclear SMAD2 3 signaling pathway: negative regulation of cell growth)

## Data Availability

All data, which was generated in the study, is contained within the article or Supplementary Material. The RetroSpect database is available at retrospect.oncobox.com. Also, data from the RetroSpect database is submitted in Zenodo (doi.org/10.5281/zenodo.7855495).

## Supplementary Materials

The following supporting information can be downloaded at: https://www.mdpi.com/article/10.3390/cells12091299/s1, Figure S1: Rank pairwise correlation between dN/dS and NGRE scores in human cell lines for 10890 genes under analysis. Figure S2: Rank pairwise correlation between dN/dS and NGRE scores in human cell lines for 710 genes under analysis. Figure S3: Intersections of GO terms for four gene groups from the whole gene set: with high both dN/dS and NGRE_AGG_ values, with high NGRE_AGG_ and low dN/dS values, with low NGRE_AGG_ and high dN/dS and with low both dN/dS and NGRE_AGG_ values. Figure S4: Intersections of GO terms for four gene groups from the 710 gene set: with high both dN/dS and NGRE_AGG_ values, with high NGRE_AGG_ and low dN/dS values, with low NGRE_AGG_ and high dN/dS and with low both dN/dS and NGRE_AGG_ values. Figure S5: Rank pairwise correlation between mean dN/dS per pathway and NPII scores for human cell lines under study. Figure S6: Intersections of GO terms for four pathway groups from the whole gene set: with high both dN/dS and NPII_AGG_ values, with high NPII_AGG_ and low dN/dS values, with low NPII_AGG_ and high dN/dS and with low both dN/dS and NPII_AGG_ values. Figure S7: Top 5 GO tags for genes included in the four groups of pathways: with high NPII_AGG_ and low dN/dS_PW_ (34 pathways); low NPII_AGG_ and high dN/dS_PW_ (12 pathways); high NPII_AGG_ and high dN/dS_PW_ (158 pathways); low NPII_AGG_ and low dN/dS_PW_ (154 pathways). The threshold for high/low values is the top/bottom 10% of pathways sorted by a corresponding value. Figure S8: Top 5 GO tags for genes included in the four groups of pathways: with high NPII_AGG_ and low dN/dS_PW_ (17 pathways); low NPII_AGG_ and high dN/dS_PW_ (2 pathways); high NPII_AGG_ and high dN/dS_PW_ (83 pathways); low NPII_AGG_ and low dN/dS_PW_ (85 pathways). The threshold for high/low values is the top/bottom 5% of pathways sorted by a corresponding value. Figure S9: Top 5 GO tags for genes included in the four groups of pathways: with high NPII_AGG_ and low dN/dS_PW_ (1 pathway); low NPII_AGG_ and high dN/dS_PW_ (1 pathway); high NPII_AGG_ and high dN/dS_PW_ (19 pathways); low NPII_AGG_ and low dN/dS_PW_ (20 pathways). The threshold for high/low values is top/bottom 1% of pathways sorted by a corresponding value. Figure S10: Distribution of NPIIAGG values (A) and dN/dSPW values (B) for 2972 human molecular pathways. Color markers define six major cancer pathways under investigation. The dashed line on panel B shows the overlap of Notch Signaling and WNT pathways. Black arrows show the median for the distributions. Figure S11: Distributions of NGRE_AGG_ and dN/dS values of genes participating in six core cancer pathways among all genes under analysis. Vertical red color lines indicate genes of the corresponding pathway of interest. Figure S12: (A) Evolutionary chart of human regulatory axis of AKT/mTOR/EGFR signaling. Color intensity reflects NGRE_AGG_ value, size of nodes is proportionate to averaged dN/dS score. Nodes where molecular information is missing are given in rhombic shape. (B) Evolutionary chart of human regulatory axis of Notch/WNT/Hedgehog signaling. Color intensity reflects NGRE_AGG_ value, size of nodes is proportionate to averaged dN/dS score. Nodes where molecular information is missing are given in rhombic shape. Table S1: The histone modifications used and their role in the regulation of transcription, Table S2: Weight coefficients for calculating aggregated NGRE and NPII values. Table S3: NGRE and NPII tissue-independent values. Table S4: *p*-values and adjusted *p*-values (Benjamini-Hohberg adjustment) for testing if NGE distribution for a gene among cell lines is higher or lower than average NGRE in all genes and cell lines. Table S5: Four gene groups from the whole gene set: with high both dN/dS and NGRE_AGG_ values, with high NGRE_AGG_ and low dN/dS values, with low NGRE_AGG_ and high dN/dS and with low both dN/dS and NGRE_AGG_ values. Table S6: GO enrichment analysis for four gene groups from the whole gene set: with high both dN/dS and NGRE_AGG_ values, with high NGRE_AGG_ and low dN/dS values, with low NGRE_AGG_ and high dN/dS and with low both dN/dS and NGRE_AGG_ values. Table S7: Four gene groups from the 710-genes set: with high both dN/dS and NGRE_AGG_ values, with high NGRE_AGG_ and low dN/dS values, with low NGRE_AGG_ and high dN/dS and with low both dN/dS and NGRE_AGG_ values. Table S8: GO enrichment analysis for four gene groups from the 710-genes set: with high both dN/dS and NGRE_AGG_ values, with high NGRE_AGG_ and low dN/dS values, with low NGRE_AGG_ and high dN/dS and with low both dN/dS and NGRE_AGG_ values. Table S9: Pathway intersections: high dN/dS, high NPII; low dN/dS, high NPII; high dN/dS, low NPII; low dN/dS, low NPII. The threshold for high/low values is top/bottom 20% of pathways sorted by a corresponding value. Table S10: GO enrichment analysis for four groups of molecular pathways: with high both dN/dS and NPII_AGG_ values, with high NPII_AGG_ and low dN/dS values, with low NPII_AGG_ and high dN/dS and with low both dN/dS and NPII_AGG_ values. Table S11: Pathway intersections: high dN/dS, high NPII; low dN/dS, high NPII; high dN/dS, low NPII; low dN/dS, low NPII. GO enrichment analysis for each group. The threshold for high/low values is the top/bottom 10% of pathways sorted by a corresponding value. Table S12: Pathway intersections: high dN/dS, high NPII; low dN/dS, high NPII; high dN/dS, low NPII; low dN/dS, low NPII. GO enrichment analysis for each group. The threshold for high/low values is the top/bottom 5% of pathways sorted by a corresponding value. Table S13: Pathway intersections: high dN/dS, high NPII; low dN/dS, high NPII; high dN/dS, low NPII; low dN/dS, low NPII. GO enrichment analysis for each group. The threshold for high/low values is top/bottom 1% of pathways sorted by a corresponding value. Table S14: Rank correlation for mean per node NGRE in six cancer-related pathways: AKT, mTOR, EGFR signaling, and Notch, WNT, Hedgehog. Table S15: Gene content of nodes for the cancer-related pathways.

## References

[1] McGrath C. (2020). Human Genetics: A Look in the Mirror. Genome Biol. Evol..

[2] Hill M.S., Vande Zande P., Wittkopp P.J. (2021). Molecular and evolutionary processes generating variation in gene expression. Nat. Rev. Genet..

[3] Peter I.S., Davidson E.H. (2011). Evolution of gene regulatory networks controlling body plan development. Cell.

[4] Anisimova A.S., Meerson M.B., Gerashchenko M.V., Kulakovskiy I.V., Dmitriev S.E., Gladyshev V.N. (2020). Multifaceted deregulation of gene expression and protein synthesis with age. Proc. Natl. Acad. Sci. USA.

[5] Kinzina E.D., Podolskiy D.I., Dmitriev S.E., Gladyshev V.N. (2019). Patterns of Aging Biomarkers, Mortality, and Damaging Mutations Illuminate the Beginning of Aging and Causes of Early-Life Mortality. Cell Rep..

[6] Suntsova M.V., Buzdin A.A. (2020). Differences between human and chimpanzee genomes and their implications in gene expression, protein functions and biochemical properties of the two species. BMC Genom..

[7] Kryazhimskiy S., Plotkin J.B. (2008). The Population Genetics of dN/dS. PLoS Genet..

[8] Whitehead A., Crawford D.L. (2006). Neutral and adaptive variation in gene expression. Proc. Natl. Acad. Sci. USA.

[9] Nikitin D., Penzar D., Garazha A., Sorokin M., Tkachev V., Borisov N., Poltorak A., Prassolov V., Buzdin A.A. (2018). Profiling of human molecular pathways affected by retrotransposons at the level of regulation by transcription factor proteins. Front. Immunol..

[10] Schumann G.G., Gogvadze E.V., Osanai-Futahashi M., Kuroki A., Münk C., Fujiwara H., Ivics Z., Buzdin A.A. (2010). Unique Functions of Repetitive Transcriptomes. Int. Rev. Cell Mol. Biol..

[11] Gogvadze E., Buzdin A. (2009). Retroelements and their impact on genome evolution and functioning. Cell. Mol. Life Sci..

[12] Nefedova L.N., Kim A. (2021). The role of retroelements in the evolution of animal genomes. Zhurnal Obs. Biol..

[13] Nikitin D., Kolosov N., Murzina A., Pats K., Zamyatin A., Tkachev V., Sorokin M., Kopylov P., Buzdin A. (2019). Retroelement-Linked H3K4me1 Histone Tags Uncover Regulatory Evolution Trends of Gene Enhancers and Feature Quickly Evolving Molecular Processes in Human Physiology. Cells.

[14] Yi S.V., Goodisman M.A.D. (2021). The impact of epigenetic information on genome evolution. Philos. Trans. R. Soc. Lond. B Biol. Sci..

[15] Simon A.J., Morrow B.R., Ellington A.D. (2018). {Retroelement-Based} Genome Editing and Evolution. ACS Synth. Biol..

[16] Yan F., Yu X., Duan Z., Lu J., Jia B., Qiao Y., Sun C., Wei C. (2019). Discovery and characterization of the evolution, variation and functions of diversity-generating retroelements using thousands of genomes and metagenomes. BMC Genom..

[17] O’Connor T., Bodén M., Bailey T.L. (2017). {CisMapper}: Predicting regulatory interactions from transcription factor {ChIP-seq} data. Nucleic Acids Res..

[18] Sadakierska-Chudy A., Filip M. (2015). A comprehensive view of the epigenetic landscape. Part {II}: Histone post-translational modification, nucleosome level, and chromatin regulation by {ncRNAs}. Neurotox. Res..

[19] Igolkina A.A., Zinkevich A., Karandasheva K.O., Popov A.A., Selifanova M.V., Nikolaeva D., Tkachev V., Penzar D., Nikitin D.M., Buzdin A. (2019). H3K4me3, H3K9ac, H3K27ac, H3K27me3 and H3K9me3 Histone Tags Suggest Distinct Regulatory Evolution of Open and Condensed Chromatin Landmarks. Cells.

[20] Nikitin D., Garazha A., Sorokin M., Penzar D., Tkachev V., Markov A., Gaifullin N., Borger P., Poltorak A., Buzdin A. (2019). Retroelement—Linked Transcription Factor Binding Patterns Point to Quickly Developing Molecular Pathways in Human Evolution. Cells.

[21] Scally A., Dutheil J.Y., Hillier L.W., Jordan G.E., Goodhead I., Herrero J., Hobolth A., Lappalainen T., Mailund T., Marques-Bonet T. (2012). Insights into hominid evolution from the gorilla genome sequence. Nature.

[22] Liu W., Wang J., Wang T., Xie H. (2014). Construction and Analyses of Human Large-Scale Tissue Specific Networks. PLoS ONE.

[23] Danino Y.M., Even D., Ideses D., Juven-Gershon T. (2015). The core promoter: At the heart of gene expression. Biochim. Biophys. Acta.

[24] Zolotovskaia M.A., Tkachev V.S., Guryanova A.A., Simonov A.M., Raevskiy M.M., Efimov V.V., Wang Y., Sekacheva M.I., Garazha A.V., Borisov N.M. (2022). OncoboxPD: Human 51 672 molecular pathways database with tools for activity calculating and visualization. Comput. Struct. Biotechnol. J..

[25] R Core Team (2018). R: A Language and Environment for Statistical Computing.

[26] Wei T., Simko V. R Package “Corrplot”: Visualization of a Correlation Matrix. https://github.com/taiyun/corrplot.

[27] Schaefer C.F., Anthony K., Krupa S., Buchoff J., Day M., Hannay T., Buetow K.H. (2009). PID: The Pathway Interaction Database. Nucleic Acids Res..

[28] QIAGEN—Pathway-Central. https://www.qiagen.com/us/shop/genes-and-pathways/pathway-central/.

[29] Fabregat A., Sidiropoulos K., Garapati P., Gillespie M., Hausmann K., Haw R., Jassal B., Jupe S., Korninger F., McKay S. (2016). The Reactome pathway Knowledgebase. Nucleic Acids Res..

[30] Nishimura D. (2001). BioCarta. Biotech Softw. Internet Rep..

[31] Kanehisa M., Goto S. (2000). KEGG: Kyoto encyclopedia of genes and genomes. Nucleic Acids Res..

[32] Romero P., Wagg J., Green M.L., Kaiser D., Krummenacker M., Karp P.D. (2004). Computational prediction of human metabolic pathways from the complete human genome. Genome Biol..

[33] Otasek D., Morris J.H., Bouças J., Pico A.R., Demchak B. (2019). Cytoscape Automation: Empowering workflow-based network analysis. Genome Biol..

[34] Shannon P., Markiel A., Ozier O., Baliga N.S., Wang J.T., Ramage D., Amin N., Schwikowski B., Ideker T. (2003). Cytoscape: A software environment for integrated models of biomolecular interaction networks. Genome Res..

[35] Saxton R.A., Sabatini D.M. (2017). mTOR Signaling in Growth, Metabolism, and Disease. Cell.

[36] Li X., Wu C., Chen N., Gu H., Yen A., Cao L., Wang E., Wang L. (2016). PI3K/Akt/mTOR signaling pathway and targeted therapy for glioblastoma. Oncotarget.

[37] Treda C., Popeda M., Ksiazkiewicz M., Grzela D.P., Walczak M.P., Banaszczyk M., Peciak J., Stoczynska-Fidelus E., Rieske P. (2016). EGFR Activation Leads to Cell Death Independent of PI3K/AKT/mTOR in an AD293 Cell Line. PLoS ONE.

[38] Edeling M., Ragi G., Huang S., Pavenstädt H., Susztak K. (2016). Developmental signalling pathways in renal fibrosis: The roles of Notch, Wnt and Hedgehog. Nat. Rev. Nephrol..

[39] Kumar V., Vashishta M., Kong L., Wu X., Lu J.J., Guha C., Dwarakanath B.S. (2021). The Role of Notch, Hedgehog, and Wnt Signaling Pathways in the Resistance of Tumors to Anticancer Therapies. Front. Cell Dev. Biol..

[40] Stefani C., Miricescu D., Stanescu-Spinu I.I., Nica R.I., Greabu M., Totan A.R., Jinga M. (2021). Growth Factors, PI3K/AKT/mTOR and MAPK Signaling Pathways in Colorectal Cancer Pathogenesis: Where Are We Now?. Int. J. Mol. Sci..

[41] Kamdje A.H.N., Kamga P.T., Simo R.T., Vecchio L., Etet P.F.S., Muller J.M., Bassi G., Lukong E., Goel R.K., Amvene J.M. (2017). Developmental pathways associated with cancer metastasis: Notch, Wnt, and Hedgehog. Cancer Biol. Med..

[42] DuBridge R.B., Tang P., Hsia H.C., Leong P.M., Miller J.H., Calos M.P. (1987). Analysis of mutation in human cells by using an Epstein-Barr virus shuttle system. Mol. Cell. Biol..

[43] Murtagh F., Legendre P. (2014). Ward’s Hierarchical Agglomerative Clustering Method: Which Algorithms Implement Ward’s Criterion?. J. Classif..

[44] Hollingsworth M.A., Swanson B.J. (2004). Mucins in cancer: Protection and control of the cell surface. Nat. Rev. Cancer.

[45] Roy L.D., Sahraei M., Subramani D.B., Besmer D., Nath S., Tinder T.L., Bajaj E., Shanmugam K., Lee Y.Y., Hwang S.I.L. (2010). MUC1 enhances invasiveness of pancreatic cancer cells by inducing epithelial to mesenchymal transition. Oncogene.

[46] Williams M.J., Zapata L., Werner B., Barnes C.P., Sottoriva A., Graham T.A. (2020). Measuring the distribution of fitness effects in somatic evolution by combining clonal dynamics with dN/dS ratios. eLife.

[47] Zapata L., Pich O., Serrano L., Kondrashov F.A., Ossowski S., Schaefer M.H. (2018). Negative selection in tumor genome evolution acts on essential cellular functions and the immunopeptidome. Genome Biol..

