# Supplementary material for: Distinct Traits of Structural and Regulatory Evolutional Conservation of Human Genes with Specific Focus on Major Cancer Molecular Pathways
Source: Cells. 2023 May 2;12(9):1299. doi: 10.3390/cells12091299 (PMC10177184; doi:10.3390/cells12091299)
Supplement: Supplementary file 1 [file cells-12-01299-s001.zip › Supplementary Figures.pdf]

Supplementary Figures

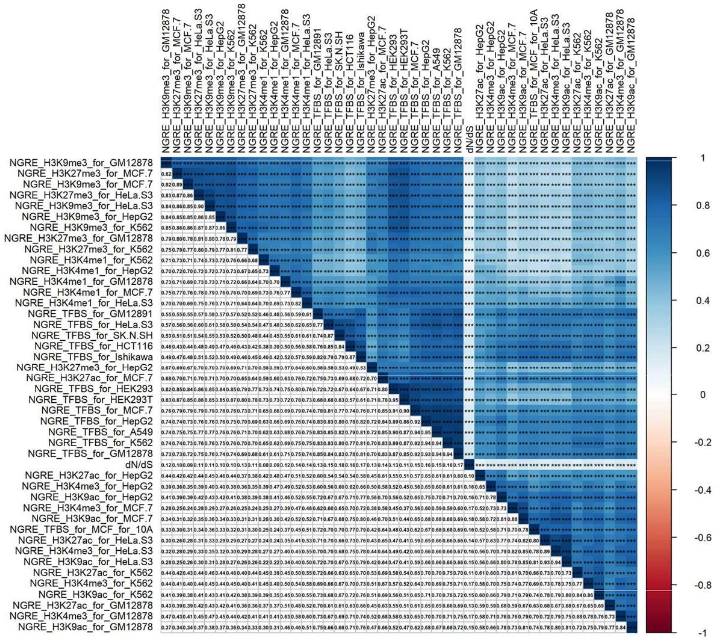

Figure S1. Rank pairwise correlation between dN/dS and NGRE scores in. human cell lines for 10890 genes under analysis. Upper matrix triangle and main diagonal are colored by rank correlation value, and asterisks denote pvalue: * stands for p<0.05, ** for p<0.01, and *** for p<0.001. Lower matrix triangle contains rank correlation coefficients in numeric format. The clustering method is Ward.D2 [43].

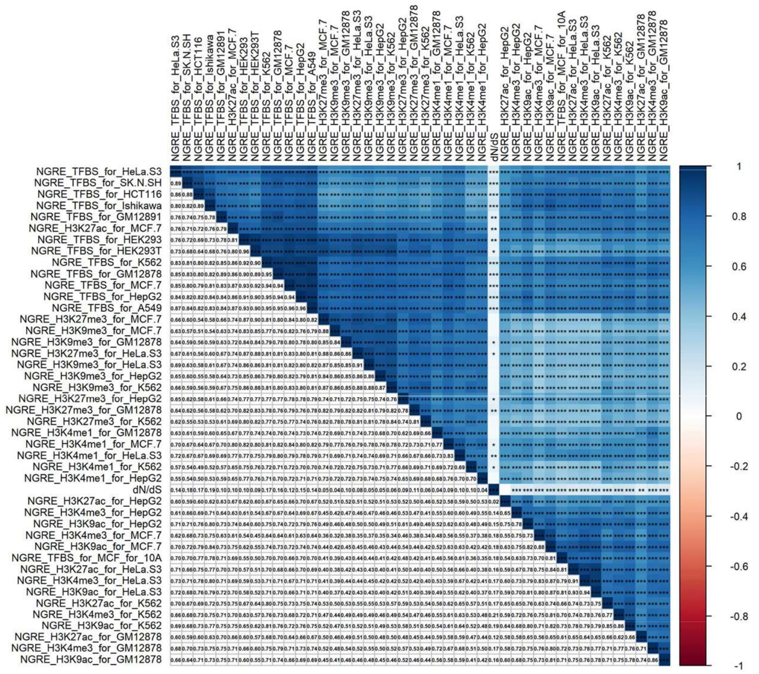

Figure S2. Rank pairwise correlation between dN/dS and NGRE scores in human cell lines for 710 genes under analysis. Correlation was calculated using 10890-gene profiles. Upper matrix triangle and main diagonal are colored by rank correlation value, and asterisks denote p-value: * stands for p<0.05, ** for p<0.01, and *** for p<0.001. Lower matrix triangle contains rank correlation coefficients in numeric format. The clustering method is Ward.D2 [43].

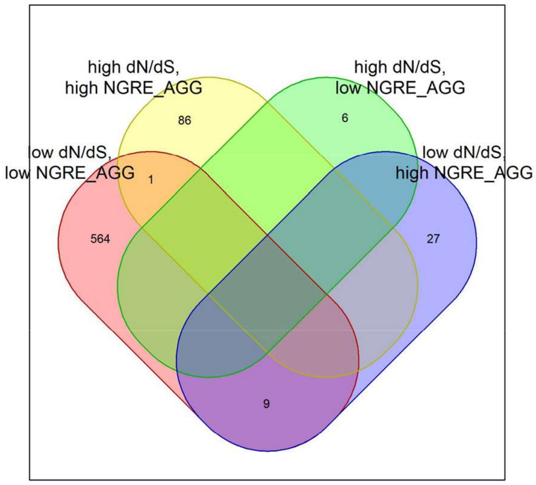

Figure S3. Intersections of GO terms for four gene groups from the whole gene set: with the high both dN/dS and $NGRE_{AGG}$ values, with the high $NGRE_{AGG}$ and low dN/dS values, with low $NGRE_{AGG}$ and high dN/dS and with the low both dN/dS and $NGRE_{AGG}$ values.

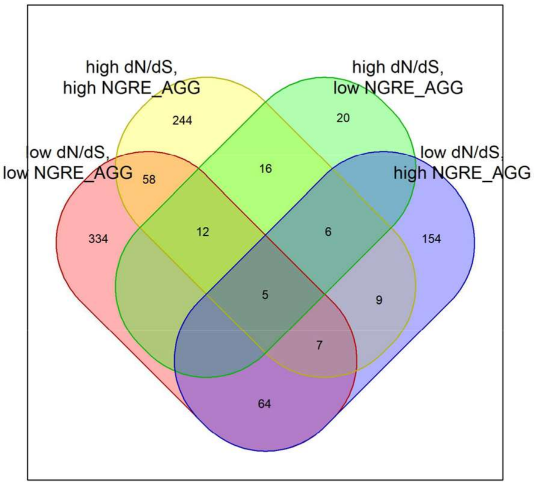

Figure S4. Intersections of GO terms for four gene groups from the 710 gene set: with the high both dN/dS and $NGRE_{AGG}$ values, with the high $NGRE_{AGG}$ and low dN/dS values, with low $NGRE_{AGG}$ and high dN/dS and with the low both dN/dS and $NGRE_{AGG}$ values.

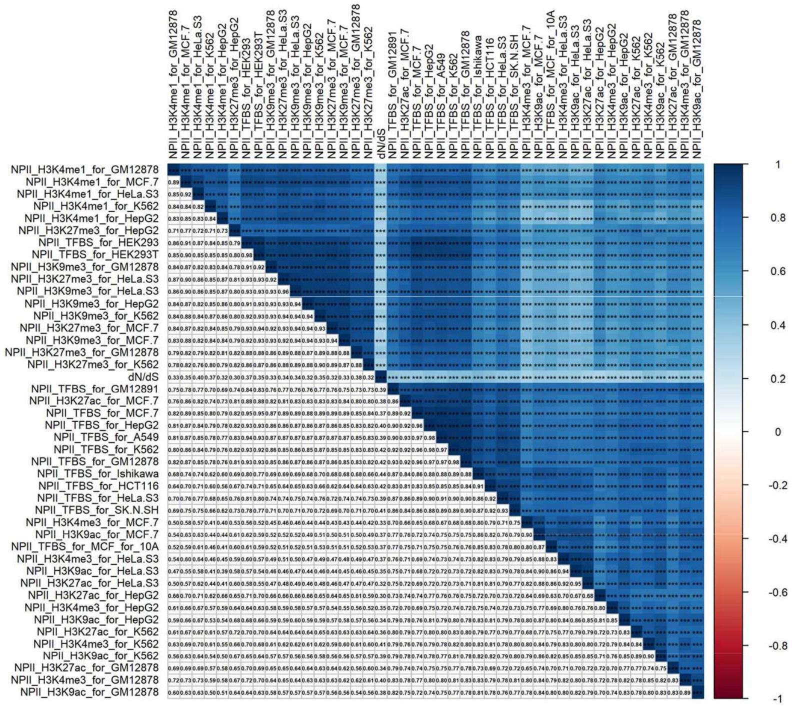

Figure S5. Rank pairwise correlation between mean dN/dS per pathway and NPII scores for human cell lines under study. Correlation was calculated using 10890-gene profiles. Upper matrix triangle and main diagonal are colored by rank correlation value, and asterisks denote p-value: * stands for p<0.05, ** for p<0.01, and *** for p<0.001. Lower matrix triangle contains rank correlation coefficients in numeric format. The clustering method is Ward.D2 [43].

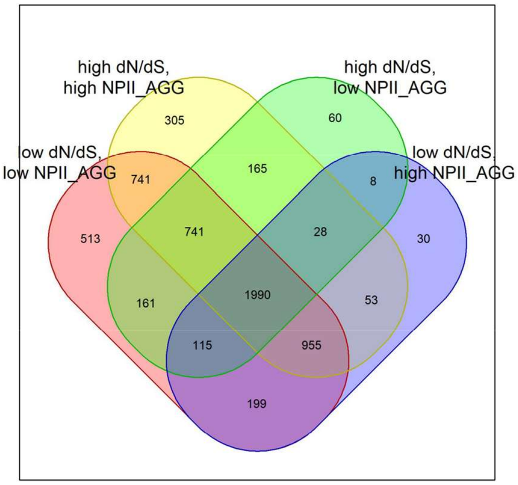

Figure S6. Intersections of GO terms for four pathway groups from the whole gene set: with the high both dN/dS and $NPII_{AGG}$ values, with the high $NPII_{AGG}$ and low dN/dS values, with low $NPII_{AGG}$ and high dN/dS and with the low both dN/dS and $NPII_{AGG}$ values.

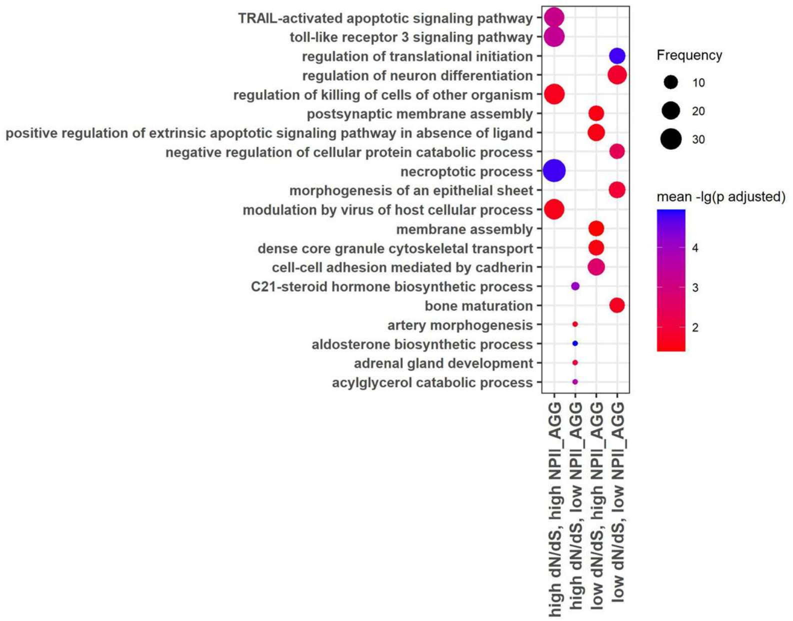

Figure S7. Top-5 GO tags for genes included in the four groups of pathways: with high $NPII_{AGG}$ and low dN/dS_{PW} (34 pathways); low $NPII_{AGG}$ and high dN/dS_{PW} (12 pathways); high $NPII_{AGG}$ and high dN/dS_{PW} (158 pathways); low $NPII_{AGG}$ and low dN/dS_{PW} (154 pathways). The threshold for high/low values is top/bottom 10% of pathways sorted by a corresponding value.

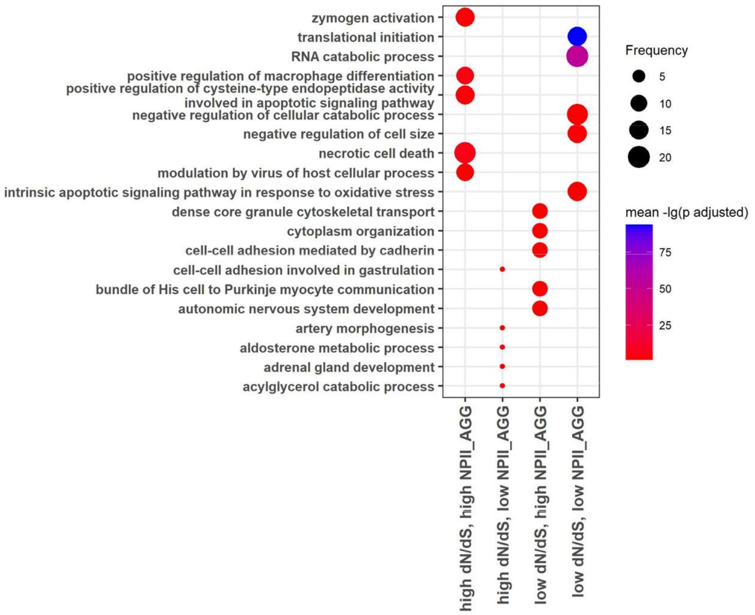

Figure S8. Top-5 GO tags for genes included in the four groups of pathways: with high $NPII_{AGG}$ and low dN/dS_{PW} (17 pathways); low $NPII_{AGG}$ and high dN/dS_{PW} (2 pathways); high $NPII_{AGG}$ and high dN/dS_{PW} (83 pathways); low $NPII_{AGG}$ and low dN/dS_{PW} (85 pathways). The threshold for high/low values is top/bottom 5% of pathways sorted by a corresponding value.

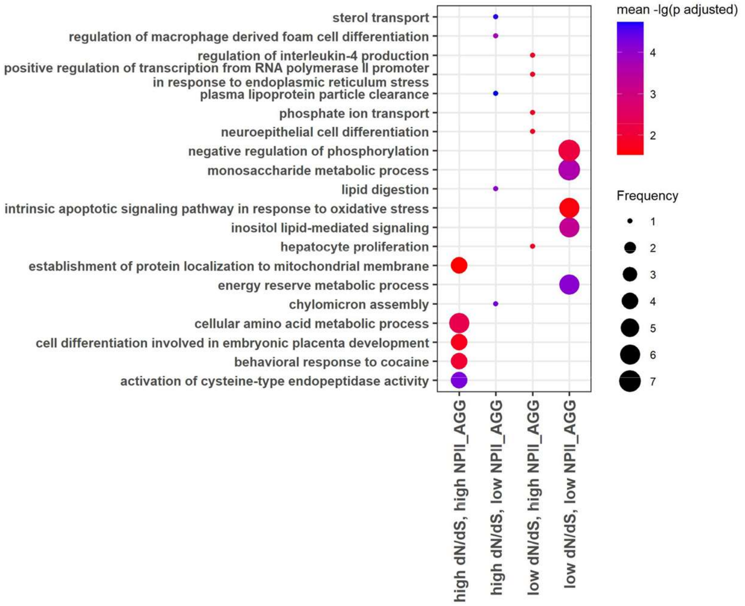

Figure S9. Top-5 GO tags for genes included in the four groups of pathways: with high $NPII_{AGG}$ and low dN/dS_{PW} (1 pathway); low $NPII_{AGG}$ and high dN/dS_{PW} (1 pathway); high $NPII_{AGG}$ and high dN/dS_{PW} (19 pathways); low $NPII_{AGG}$ and low dN/dS_{PW} (20 pathways). The threshold for high/low values is top/bottom 1% of pathways sorted by a corresponding value.

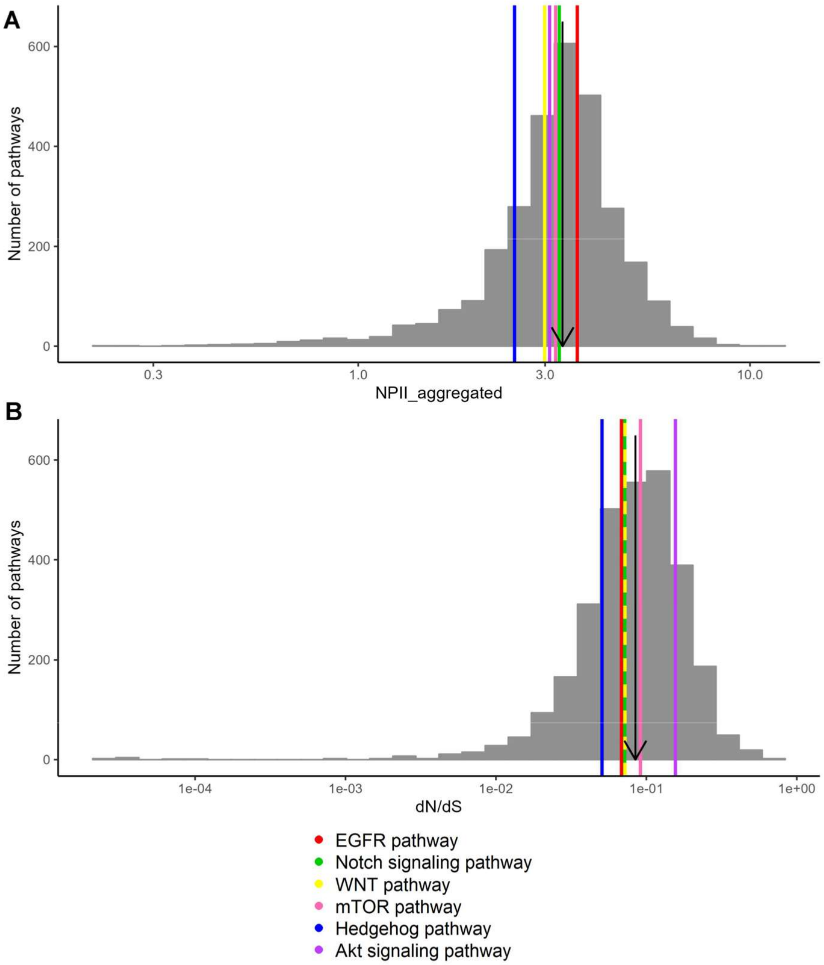

Figure S10. Distribution of NPIIAGG values (A) and dN/dSPW values (B) for 2972 human molecular pathways. Color markers define six major cancer pathways under investigation. The dashed line on panel B shows overlap of Notch Signaling and WNT pathways. Black arrows show median for the distributions. .

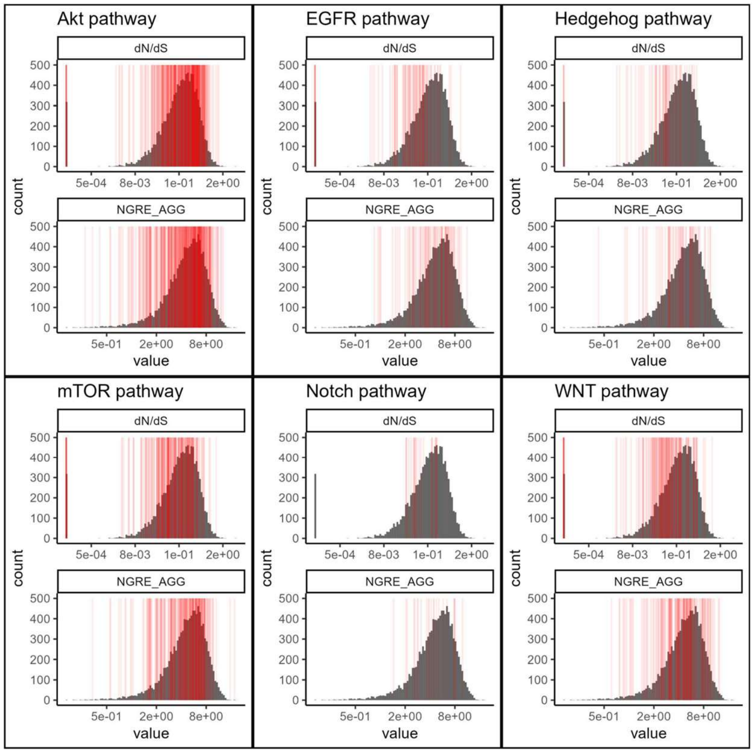

Figure S11. Distributions of NGRE$_{AGG}$ and dN/dS values of genes participating in six core cancer pathways among all genes under analysis. Vertical red color lines indicate genes of the corresponding pathway of interest. .

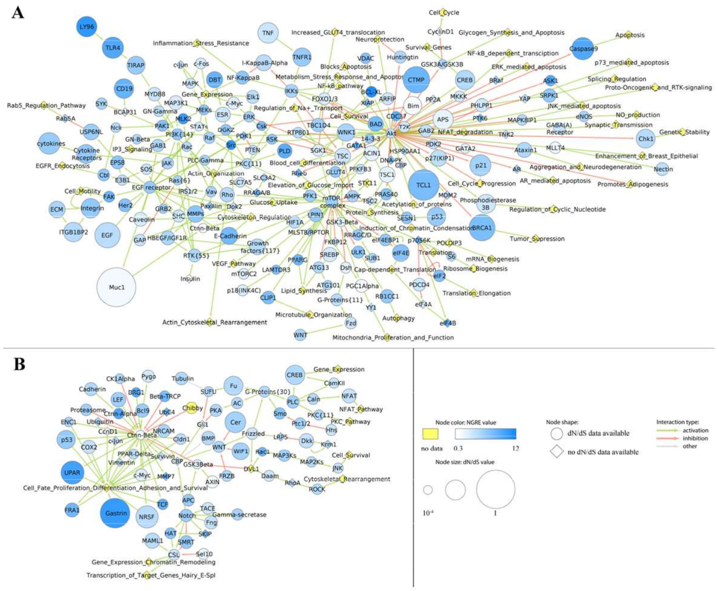

Figure S12. (A) Evolutionary chart of human regulatory axis of AKT/mTOR/EGFR signaling. Color intensity reflects NGRE_{AGG} value, size of nodes is proportionate to averaged dN/dS score. Nodes where molecular information is missing are given in rhombic shape. (B) Evolutionary chart of of human regulatory axis of Notch/WNT/Hedgehog signaling. Color intensity reflects NGRE_{AGG} value, size of nodes is proportionate to averaged dN/dS score. Nodes where molecular information is missing are given in rhombic shape. .
